# Assembly of glioblastoma tumoroids and cerebral organoids: a 3D *in vitro* model for tumor cell invasion

**DOI:** 10.1002/1878-0261.13740

**Published:** 2024-10-30

**Authors:** Jieun Kim, Rokhyun Kim, Wonseok Lee, Gyu Hyun Kim, Seeun Jeon, Yun Jin Lee, Jong Seok Lee, Kyung Hyun Kim, Jae‐Kyung Won, Woochan Lee, Kyunghyuk Park, Hyun Je Kim, Sun‐Wha Im, Kea Joo Lee, Chul‐Kee Park, Jong‐Il Kim, Ji Yeoun Lee

**Affiliations:** ^1^ Department of Anatomy and Cell Biology Seoul National University College of Medicine Seoul Korea; ^2^ Medical Research Center Genomic Medicine Institute, Seoul National University Seoul Korea; ^3^ Department of Biomedical Sciences Seoul National University College of Medicine Seoul Korea; ^4^ Department of Transitional Medicine Seoul National University College of Medicine Seoul Korea; ^5^ Department of Neurosurgery, Seoul National University Hospital Seoul National University College of Medicine Seoul Korea; ^6^ Laboratory of Synaptic Circuit Plasticity, Neural Circuits Research Group Korea Brain Research Institute Daegu Korea; ^7^ Division of Pediatric Neurosurgery Seoul National University Children's Hospital Seoul Korea; ^8^ Department of Pathology, Seoul National University Hospital Seoul National University College of Medicine Seoul Korea; ^9^ Cancer Research Institute, Medical Research Center Seoul National University College of Medicine Seoul Korea; ^10^ Department of Biochemistry and Molecular Biology Kangwon National University School of Medicine Chuncheon Korea; ^11^ Department of Biochemistry and Molecular Biology Seoul National University College of Medicine Seoul Korea; ^12^ Neuroscience Research Institute, Medical Research Center Seoul National University College of Medicine Seoul Korea

**Keywords:** assembloid, organoid, glioblastoma, invasion, tumor microtube

## Abstract

Glioblastoma (GBM) has a fatal prognosis because of its aggressive and invasive characteristics. Understanding the mechanism of invasion necessitates an elucidation of the relationship between tumor cells and the tumor microenvironment. However, there has been a scarcity of suitable models to investigate this. In this study, we established a glioblastoma‐cerebral organoid assembloid (GCOA) model by co‐culturing patient‐derived GBM tumoroids and human cerebral organoids. Tumor cells from the tumoroids infiltrated the cerebral organoids, mimicking the invasive nature of the parental tumors. Using time‐lapse imaging, various invasion patterns of cancer cells within cerebral organoids resembling a normal tissue milieu were monitored. Both single‐ and collective‐cell invasion was captured in real‐time. We also confirmed the formation of an intercellular tumor network and tumor–normal‐cell interactions. Furthermore, the transcriptomic characterization of GCOAs revealed distinct features of invasive tumor cells. Overall, this study established the GCOA as a three‐dimensional (3D) *in vitro* assembloid model to investigate invasion mechanisms and interactions between tumor cells and their microenvironment.

## Graphical abstract

This study presents a glioblastoma‐cerebral organoid assembloid (GCOA) model to investigate tumor cell invasion. The GCOA integrates patient‐derived GBM tumoroids with cerebral organoids, enabling real‐time 3D imaging of tumor cell behavior, intercellular interactions, and gene expression differences between invading and non‐invading tumor cells.

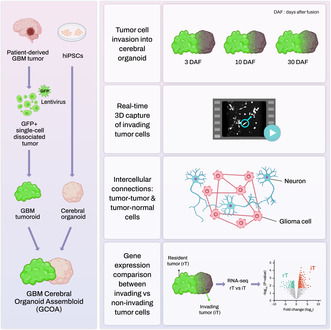

**Abbreviations**
CCRT: concurrent chemoradiotherapy
CDH1: cadherin 1
CDKN1C: cyclin‐dependent kinase inhibitor 1C
CLEM: correlative light and electron microscopy
CO: cerebral organoid
COL3A1: collagen type 3 alpha 1 chain
CPM: counts per million
Cx43: connexin 43
DAF: days after fusion
DCN: decorin
DEG: differentially expressed gene
DIV: days in vitro
DLK1: delta like non‐canonical notch ligand 1
GAP43: growth associated protein 43
GBM: glioblastoma
GCOA: glioblastoma‐cerebral organoid assembloid
GFP: green fluorescent protein
GLUR1: glutamate receptor 1
GO: gene ontology
HCS: high‐content imaging system
IHC: immunohistochemistry
IT: invading tumor cells
ITGA1: integrin subunit alpha 1
MRI: magnetic resonance imaging
NGS: neurogliomal synapse
NPC: neural progenitor cell
NPY: neuropeptide Y
PCA: principal component analysis
PLAU: plasminogen activator urokinase
RIN: RNA integrity number
RT: resident tumor cells
SEM: scanning electron microscopy
SNAI1: Snail family transcriptional repressor 1
TME: tumor microenvironment
TM: tumor microtube
TNC: tenascin C
UMI: unique molecular identifier
VGLUT1: vesicular glutamate transporter 1
VIM: vimentin

## 1 Introduction

Glioblastoma (GBM) is the most common primary malignant brain tumor in adults and has a fatal prognosis [1]. The median survival time is approximately 15 months with standard surgical resection followed by concurrent chemoradiotherapy (CCRT) [2]. The invasive behavior of GBM is a major underlying factor in its aggressive clinical course and poor prognosis, as diffuse microinvasion, even in distant regions of the brain, occurs in the initial stage of the disease [3, 4, 5]. Experiments have revealed various aspects of GBM invasion, highlighting the significance of the intrinsic properties of tumor cells and triggers from the tumor microenvironment (TME) [6, 7, 8]. Moreover, genomic analyses of tumor tissues have identified tumor subtypes related to invasion and revealed critical pathways involved in the invasion process [9, 10]. Despite significant advancements in our knowledge of the invasion mechanisms of GBM, no approved treatment that directly inhibits invasion exists [6].

Various models have been developed to study GBM invasion, including 2D cell line cultures, 3D cultures of patient‐derived glioma stem cells (GSCs), and patient‐derived xenografts [11, 12]. Although these models have proven valuable in previous studies, they have significant limitations. For instance, 2D models cannot mimic the intra‐tumor heterogeneity of GBM and the interaction of tumor cells with the TME [13]. Although GSCs facilitate long‐term cultures of tumor cells in 3D structures [14], they cannot preserve complex tissue structures, such as the extracellular matrix and TME. *In vivo* mouse models have been established to address the complexities associated with the environmental influences on GBM research [15, 16]. However, these animal models have limitations as well: they are time‐consuming and expensive to maintain, and they cannot mimic the human TME and replicate the heterogeneity of human tumors, owing to inherent species differences [15, 16]. Therefore, the development of a model that can accurately recapitulate human GBM characteristics and the TME is immensely valuable for elucidating the biology of GBM invasion.

Organoids have recently emerged as promising tools for modeling human organ development and pathology *in vitro* [17]. These 3D structures can reflect the key elements and functional properties of various organs [18]. Cerebral organoids (COs) have exhibited considerable potential for mimicking important aspects of human brain development, including corticogenesis, gyrification, and synaptogenesis, thus enhancing our understanding of neurological disorders [19]. Although COs resemble the normal brain, they lack cells of non‐epithelial origin, such as immune cells and vasculature [20]. Therefore, COs can serve as a partial recapitulation model for the TME.

Tumoroids, also known as tumor organoids, have also gained recognition as valuable models for oncology research. Tumoroids maintain the heterogeneity and genomic/molecular features of their parent tumors, making them a relevant representation of the disease [21]. Multiple GBM models have been described in the literature [22]. For instance, a neoplastic CO model replicated GBM tumorigenesis through the genetic manipulation of COs [23, 24]. Although this model mimics the natural progression of GBM, it represents only a limited range of mutations and oncogenes [23, 24]. GBM tumoroids generated from patient tumor tissues have successfully replicated the histological features, tumor heterogeneity, gene expression, and mutational profiles of the corresponding original tumors [25, 26] [Correction added on 26 February 2025 after first online publication: The term “GBM tumors” has been replaced with “GBM tumoroids.”]. Although these tumoroid models are valuable for studying GBM, they face challenges when exploring the mechanisms underlying tumor invasion into the surrounding normal brain tissue and the interactions between normal and tumor cells [25, 26]. Recent studies have investigated GBM behavior within a more relevant brain‐like environment by co‐culturing patient‐derived tumor cells with COs [27, 28]. However, these studies encountered limitations because the primarily cultured tumor cells lacked intratumoral heterogeneity.

To further enhance modeling capabilities, we developed an assembloid model by fusing a patient‐derived GBM tumoroid with a human induced pluripotent stem cell (hiPSC)‐derived CO. This model, termed the GBM tumoroid‐cerebral organoid assembloid (GCOA), utilizes GBM tumoroids to maintain intra‐tumor heterogeneity and COs to partially recapitulate the surrounding normal brain tissue. Using GCOAs, we elucidated various aspects of GBM invasion mechanisms, including cellular invasion patterns, tumor microtube (TM) formation, interactions with normal cells, and gene expression.

## 2 Materials and methods

### 2.1 GBM tumoroid culture

Fresh tumor tissues were obtained with written informed consent from two patients at Seoul National University Hospital (SNUH‐NG‐27 and SNUH‐NG‐65) who underwent surgical resection of newly diagnosed GBM from February 2019 to March 2020. All study methodologies were conducted in accordance with the Declaration of Helsinki. The pathogenetic and genomic information for both patients is presented in Table S1. The tissue was minced and incubated with 1 mg⋅mL^−1^ dispase II (Roche, Basel, Switzerland), 0.1 mg⋅mL^−1^ collagenase (Sigma‐Aldrich, St. Louis, MO, USA), and penicillin/streptomycin in DMEM for 15 min at 37 °C. After incubation, the samples were passed through a 50 mL syringe equipped with an 18‐G needle five times, and only the supernatant was filtered through 40 μm strainers. Single‐cell dissociated samples were incubated in RBC lysis buffer (Thermo Fisher, Waltham, MA, USA) and embedded in 20 μL of Matrigel (Corning Inc., Corning, NY, USA). GBM tumoroids were cultured in ultra‐low attachment plates (Corning Inc.) with NBM complete medium on an orbital shaker at 120 rpm [25, 29]. For passaging, tumoroid cultures were dissociated by gentle pipetting and embedded in Matrigel once the tumoroids reached over 90% confluence. Experiments involving human patient materials were performed with the approval of the Institutional Review Board (IRB) of Seoul National University Hospital (#C‐2009‐020‐1156).

### 2.2 Lentiviral labeling

A GFP vector was generated by transfecting the lentiviral pLB vector into HEK293T cells using Lipofectamine 2000 (Thermo Fisher, Waltham, MA, USA) and concentrating the supernatant with a Lenti‐X concentrator (Clontech Laboratories Inc., Mountain View, CA, USA) after 72 h. GBM cells were infected with the lentivirus and incubated with polybrene diluted in NBM for 6 h, and the infected cells were embedded in Matrigel. Lentivirally labeled GBM tumoroids were maintained in NBM complete medium and passaged monthly, as described above.

### 2.3 Cerebral organoid culture

The hiPSC line CMC‐hiPSC‐009 (RRID: CVCL_WR31) was obtained from the Korea National Stem Cell Bank (nih.go.kr/ncsr). The hiPSCs were cultured under feeder‐free conditions in mTeSR Plus medium (StemCell Technologies, Vancouver, Canada) on vitronectin‐coated plates. The cell line used was initially evaluated and authenticated by short tandem repeat (STR) analysis, and its cell attachment and morphology were compared to those reported in previous literature over the past 3 years. The cell lines were regularly tested for mycoplasma contamination, and all experiments were conducted using mycoplasma‐free cells. They were passaged every 2–3 days using ReLeSR reagent (StemCell Technologies). COs were generated through the enCOR method [30] using a Cerebral Organoid Kit (Stem Cell Technologies). Briefly, 9000 dissociated hiPSCs were seeded into a single well of a 96‐well U‐bottom low‐attachment plate to form an embryoid body (EB). After 5 days *in vitro* (DIV), neural induction medium was administered to the EBs. After 2–3 days, EBs with clear neuroectodermal layers were embedded in Matrigel containing expansion medium. Embedded EBs were maintained in maturation medium on an orbital shaker at 10 DIV. COs were fed every other day with the maturation medium, and from 30 DIV onward, dissolved Matrigel (1:50) was added to the maturation medium.

### 2.4 GCOA culture

To fuse COs and GBM tumoroids together, we followed the protocol described by Pasca et al. [31]. Briefly, GBM tumoroids and COs were cultured separately and then transferred to the bottom of 1.5 mL microcentrifuge tubes in contact with each other for fusion in CO maturation medium without supplementing Matrigel. Seven days after fusion (DAF), the GCOAs were carefully transferred to 6‐well ultra‐low attachment plates using wide‐bore P1000 pipette tips. The assembly was performed using GBM tumoroids that had been passaged for approximately 1 month and COs at 40 DIV.

### 2.5 Live imaging

The movement of GFP‐positive cells in the GCOAs was imaged for 90 h under environmentally controlled conditions (37 °C, 5% CO_2_) using an ImageXpress Micro Confocal High‐Content Imaging System (Molecular Devices, San Jose, CA, USA). GCOAs were transferred to a well of a U‐bottom 96‐well plate (Corning Inc.) containing 300 μL of maturation medium. Images were acquired with 50 planes with 50‐μm Z‐stack intervals at a rate of 30 min per frame and generated as a 2D maximum projection.

### 2.6 Histological and immunohistochemical analysis

GBM tumoroids were collected simultaneously at the initiation of co‐culturing and fixed in 4% paraformaldehyde (PFA) at 4 °C overnight. Subsequently, they were embedded in paraffin and sectioned for hematoxylin and eosin (H&E) and immunohistochemical (IHC) staining. For IHC analysis, the slides were blocked and incubated with primary antibodies at 4 °C overnight, followed by incubation with horseradish peroxidase and the DAB Substrate Kit (Vector Laboratories, Newark, CA, USA). The primary antibodies used were as follows: anti‐ATRX (1:800; Abcam, Cambridge, UK), anti‐c‐MET (1:500; Thermo Fisher, Cambridge, UK), anti‐EGFR (1:1000; Abcam), anti‐GFAP (1:2000; Abcam), and anti‐IDH1‐R132H (IDH1mt) (1:500; Merck Millipore, Burlington, MA, USA). Samples were fixed and frozen for immunofluorescence (IF) staining. The slides were blocked and incubated with primary antibodies at 4 °C overnight at the following concentrations: anti‐CD3 (1:100; Novus Biologicals, St. Louis, MO, USA), anti‐CD45 (1:50; Novus Biologicals), anti‐CD68 (1:50; Santa Cruz Biotechnology Heidelberg, Germany), anti‐GAP43 (1:200; Abcam), anti‐Ki67 (1:200; BD Biosciences‐Pharmingen, Becton, NJ, USA), anti‐Nestin (1:100; Genetex, Irvine, CA, USA), anti‐NeuN (1:250; Merck Millipore), anti‐Snail (1:100; Invitrogen, Waltham, MA, USA), anti‐SOX2 (1:100; Merck Millipore), anti‐TUJ1 (1:250; Merck Millipore), and anti‐Vimentin (1:100; Abcam). This was followed by incubation with the secondary antibodies (1:500) and Alexa Fluor 633 phalloidin (1:20; Invitrogen) for F‐actin staining for 2 h at room temperature (RT). Slides were mounted after DAPI staining, and images were captured using a confocal microscope (Leica, Wetzlar, Germany).

### 2.7 Whole mount staining and clearing

We followed a published protocol from the Vienna Biocenter using ethyl cinnamate (ECi) as an index‐matching solution [32]. GCOAs were stained and cleared using the same whole‐mount staining and clearing methods as previously described [33]. The GCOAs were fixed in 4% PFA at 4 °C overnight. Following this, the GCOAs were blocked and permeabilized in a PBS‐TxDBN solution [2% Triton‐X‐100, 20% DMSO, 5% BSA, 0.05% NaN3 in PBS] at RT for 2 days on a shaker. When required, GCOAs were incubated with primary antibodies (anti‐connexin 43 (1:20; Sigma–Aldrich), anti‐GLUR1 (1:50; Merck Millipore), and anti‐VGLUT1 (1:30; Invitrogen)) in 400 μL of PBS‐TxDBN at RT for 5 days. GCOAs were then washed with PBS‐TxDBN for 2 days and subsequently treated with secondary antibodies (1:50; Invitrogen) and Alexa Fluor 633 phalloidin (1:10; Invitrogen) for F‐actin detection, along with DAPI (1:100), in 400 μL of PBS‐TxDBN at RT for 5 days. The samples were then washed with PBS‐TxDBN for 2 days and fixed with 4% PFA at 4 °C overnight. Stained GCOAs were dehydrated with serial concentrations of 30%, 50%, 70%, and 2 × 99.7% anhydrous 1‐propanol (Sigma‐Aldrich) in PBS with pH adjusted to 9.0–9.5 using trimethylamine, and incubated at 4 °C for at least 4 h per step. For refractive index matching, dehydrated GCOAs were immersed in ECi (Sigma‐Aldrich) for at least 1 h at RT prior to image acquisition. The transparent GCOAs were imaged using a confocal microscope (Leica).

### 2.8 Visualizing the invasion of tumor cells into the COs using correlative light and electron microscopy (CLEM)

GCOAs were fixed with 2% PFA and 2.5% glutaraldehyde in a 0.1 m cacodylate buffer solution (pH 7.4) at 14 DAF. Fixed GCOAs were sliced into 150 μm‐thick sections using a vibratome (#VT1000S, Leica) with agarose embedding and stained with DAPI. The ROIs were imaged using a Nikon A1R confocal microscope mounted on a Nikon Eclipse Ti‐E body. Following confocal imaging, bright‐field images were used as a reference to assist the precise dissection of the imaged regions. Dissected tissues were processed for heavy metal staining, dehydration, and resin embedding for scanning electron microscopy (SEM) as previously described [34]. Following manual trimming of the samples, serial sections were collected on carbon nanotube (CNT)‐coated PET tapes (Boeckeler Instruments, Tucson, AZ, USA) using an ATUMtome (Boeckeler Instruments) and an ultra‐Maxi knife (DiATOME, Quakertown, PA, USA). Sections on the CNT tape strips were mounted on a 4‐inch silicon wafer with a double‐sided adhesive conductive tape (Ted Pella, Redding, CA, USA). The sections were imaged using an SEM (Zeiss, Oberkochen, Germany) with either an in‐lens secondary electron detector (SE) or a backscattered electron detector (BSD). ATLAS 5 software (Fibics Incorporated, Ottawa, Canada and Zeiss) was used for large‐area imaging. Using low‐resolution images (80 nm pixel size), we correlated and confirmed the current position relative to the visible marked structures (nuclei) of the ROI. This information enabled a precise correlation between different imaging modalities in high‐resolution ROI imaging. Two hundred serial SEM images were obtained using an operating beam voltage of 5 kV and BSD detection with a dwell time of 7 μs. The pixel average was set over a pixel size of 5 × 5 nm, and each slice had a thickness of 50 nm. The acquired images were aligned using ImageJ software and the Fiji plugin TrakEM2. Based on their xyz coordinates within the ROI and using fiducials correlated between electron and confocal microscopy datasets, we accurately identified and located the GFP‐positive tumor cells of interest.

### 2.9 Real‐time qPCR

mRNA was extracted using the RNeasy Mini Kit (Qiagen, Hilden, Germany), and template cDNA was synthesized via reverse transcription using the Maxime RT PreMix Kit (iNtRON Biotechnology, Seongnam, Korea). qPCR was performed using SYBR Green (Qiagen) on a CFX Connect Machine (Bio‐Rad, Hercules, CA, USA). The primers used are listed in Table S2.

### 2.10 RNA quant sequencing and data analysis

GCOAs were co‐cultured for 2 weeks (14 DAF) and separated into two parts: COs and GBM tumoroids. The samples were processed using the ABDK program (37C_ABDK_01 for COs and 37C_ABDK_02 for GBM tumoroids) on a gentleMACS Octo Dissociator with Heaters (Miltenyi Biotec, Bergisch Gladbach, Germany) using the Adult Brain Dissociation Kit (Miltenyi Biotec) following the manufacturer's protocol. Dissociated cells were passed through a 70 μm cell strainer, resuspended in 300 μL FACS buffer (1% BSA in PBS), and loaded onto a SH800S FACS sorter (Sony, Tokyo, Japan). GFP‐positive cells were classified as resident tumors (rT) in the GBM tumoroid portion of the GCOAs, and invading tumors (iT) in the CO portion of the GCOAs when GFP‐negative cells were discarded. All experiments were performed in triplicate to determine statistical significance. RNA was extracted from rT and iT cells immediately after FACS using the RNeasy kit (Qiagen) according to the manufacturer's instructions. RNA quality was assessed with an Agilent 2100 bioanalyzer using an RNA 6000 Nano Chip (Agilent Technologies, Santa Clara, CA, USA) to determine RNA integrity number (RIN) values and concentrations. Libraries were constructed using a QuantSeq 3′‐mRNA‐Seq Library Prep Kit (Lexogen, Vienna, Austria) according to the manufacturer's instructions. The quality of the libraries was determined with an Agilent 2100 bioanalyzer using a High Sensitivity DNA Chip, and the paired‐end reads (2 × 150 bp read length) were sequenced on a HiSeq platform (Illumina, San Diego, CA, USA) to produce 2 Gb of data per sample. Raw data quality control was performed using FastQC (v0.11.9) [35] to evaluate the general mapping quality, including the number of aligned reads and the ratio of duplicate reads. The QC data are presented in Table S3. The unique molecular index (UMI) of each read was extracted using the ‘extract’ function from umi‐tools (v1.1.2) [36]. Using Illumina sequencing adapters and polyA fasta as reference files, sequencing reads were trimmed using the bbduk.sh script from BBMap software (38.87) [37]. After trimming, the reads were aligned to the human reference genome (GRCh38/hg38) using the STAR aligner (2.7.9a) [38] and Samtools (1.13 + htslib‐1.13) [39]. PCR duplicates were removed using the UMI‐tools Dedup function. To quantify gene expression, the HTSeq (v0.13.5) [40] htseq‐count function was used. All experiments were performed at the Genomic Medicine Institute Research Service Center, Seoul National University College of Medicine.

The read counts and counts per million (CPM) values of each data point were imported into R. Principal component analysis (PCA) was visualized using the autoplot and ggplot2 packages. The hierarchical clustering heatmap of the genes was visualized using the pheatmap package with Z‐scores. Differential expression analysis was performed using the DESeq2 package, with the design accounting for patient differences [41], and volcano plots of differentially expressed genes (DEGs) were visualized using the EnhancedVolcano package. Base mean and fold changes were calculated using the DESeq2 algorithm, with statistical significance defined as *P* or *P*_adj_ <0.05. Significant DEGs were identified with a base mean >5, *P* value <0.05, and log_2_(fold change) > 1. In the DEG list, we ranked significant genes using a scoring system based on the log_2_(fold change) value multiplied by the negative log *P* value. A web‐based tool, g:profiler [42], was used to evaluate the ontology and identify the underlying pathways of the top‐scoring genes. The synapse‐related genes were extracted from a study by Ji et al. [43]. Gene ontology (GO) terms and bubble plots for synapse‐related genes were plotted using https://www.bioinformatics.com.cn/en, a free online platform for data analysis and visualization.

We compared our results with those from the human GBM tissue RNA‐seq database established by the IVY‐Glioblastoma Atlas Project [44]. The DEGs from the Leading Edge and Microvascular Proliferation in the Cellular Tumor regions were obtained from the IVY Glioblastoma Atlas Project (https://glioblastoma.alleninstitute.org), and certain identified genes are discussed in the Results section. We then benchmarked the cibersortx webtool [45], an analytical tool for estimating the abundance of cell fractions within a mixed cell population. The input for the cibersortx algorithm was created using gene module expression data extracted from single‐cell data from GBM tissues [45]. Single‐cell data with metadata generated by Smart‐Seq2 [45] were downloaded from the Single Cell Portal website provided by the Broad Institute (Gene Ontology Omnibus, GSE131928). Within the single‐cell data, each barcoded cell was assigned scores for the four cellular states within the metadata. Since the GCOA samples were derived from adult patients, we excluded cells from pediatric patients. Following the scoring system employed by Neftel et al., we excluded cells with hybrid cellular states; therefore, only malignant cells with specific cellular states derived from adult patients were selected. Next, an expression matrix with the gene modules provided by Neftel et al. was created as a Seurat object for pseudobulk expression and used as the input reference gene signature mixture [45]. Quant‐seq data of GCOAs were used to estimate the fractions of their cellular states. The CIBERSORTx algorithm was run with 500 permutation parameters for statistical estimation and batch correction in B‐mode, which is recommended for the Smart‐Seq2 dataset reference. A histogram was plotted using https://www.bioinformatics.com.cn/en. All the analyses were performed using a computing server at the Genomic Medicine Institute Research Service Center.

Meanwhile, the 27iT_1 data was discarded owing to failing QC. The gene expression counts measured by HTSeq (v0.13.5) [40] for the 27iT_1 data were the lowest among all samples, with only 1/5–10 counts, compared with the second lowest count data. Details on the sequencing QC are provided in Table S3.

## 3 Results

### 3.1 Establishment of the GBM invasion model through organoid co‐culture

**Fig. 1:**
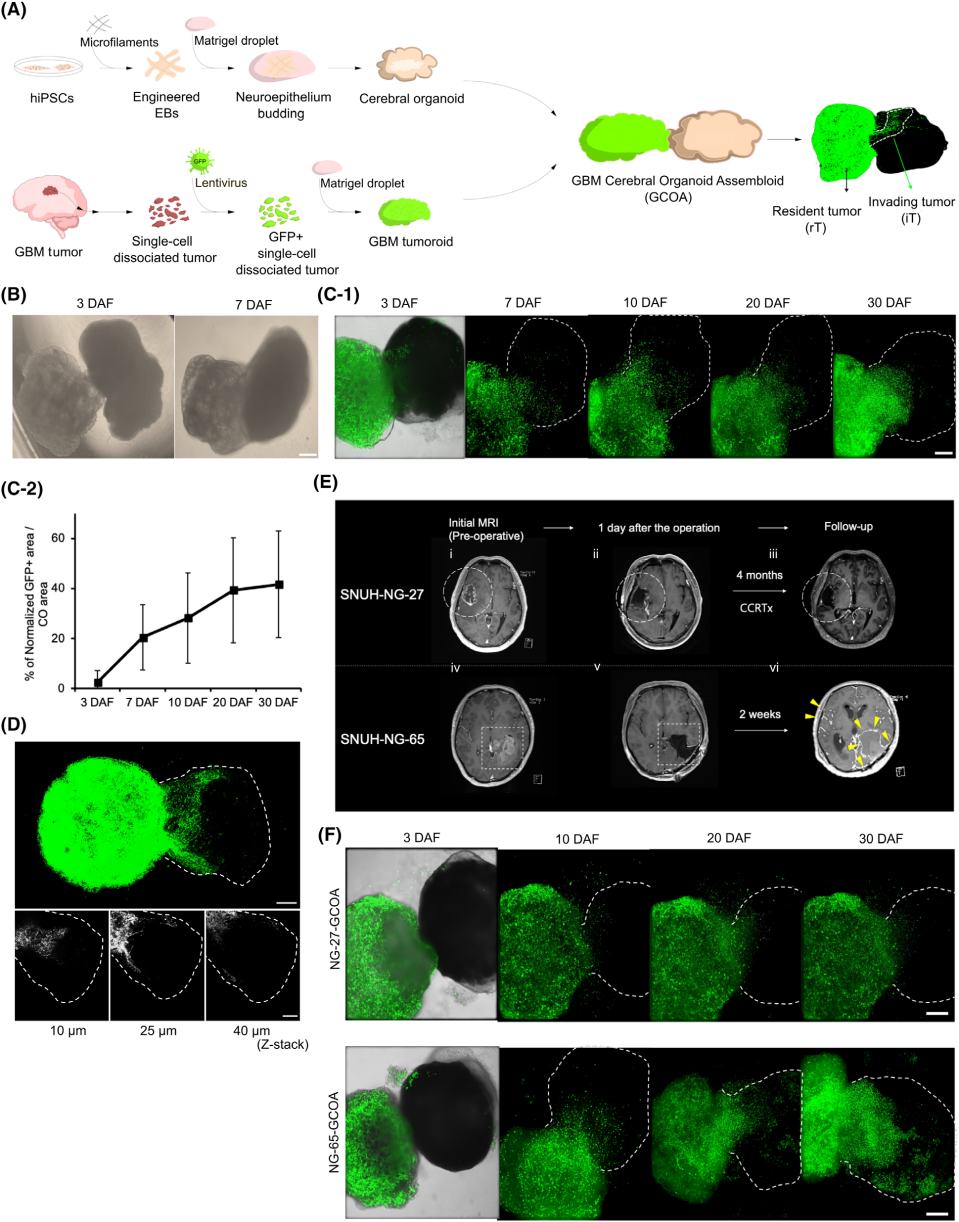
Generation of 3D GBM invasion model using assembloid culture system. (A) A Schematic diagram describing the generation of the GBM‐cerebral organoid assembloid (GCOA) by co‐culturing human induced pluripotent stem cells (hiPSCs)‐derived cerebral organoids (COs) and GFP‐labeled patient‐derived GBM tumoroids. Tumor cells that remained in the tumoroid part of GCOAs are named resident tumor cells (rT) and tumor cells infiltrating into COs are named invading tumor cells (iT). GBM, glioblastoma; EBs, embryoid bodies. (B) Morphology of a GCOA at 3 and 7 days after fusion (DAF). *n* = 10. Scale bar: 500 μm. (C‐1) Images of sequential expression of GFP fluorescence in a GCOA over 30 DAF indicating an area of a CO with dashed lines. *n* = 6. Scale bar: 500 μm. (C‐2) Line graph of relative areas of normalized (by individual initial tumoroid size) invaded GFP‐positive (GFP+) tumor cells in the CO to the total area of the CO over 30 DAF. *n* = 6; error bars represent SD. (D) Representative fluorescence images of a maximum projection and z‐stack positions of a GCOA at 14 DAF, indicating an area of a CO with dashed lines. *n* = 6. Scale bars: 200 μm. (E) MRI images of SNUH‐NG‐27 and SNUH‐NG‐65 patients: SNUH‐NG‐65 patient exhibited significantly accelerated tumor progression compared to SNUH‐NG‐27 patient. SNUH‐NG‐27 had a heterogeneously enhancing mass in the right temporal lobe on the initial MRI (i, dotted circle), and gross total resection (GTR) was performed, as no residual mass was evident on the MRI taken 1 day after the operation (ii). A follow‐up MRI was conducted 4 months after the operation with no evident mass (iii). SNUH‐NG‐65 patient initially had a heterogeneously enhancing mass in the left temporal lobe (iv, dotted square), along with another enhancing nodule in the suprasellar area (not shown). GTR of the main mass was performed (v). However, only 2 weeks after the operation, diffuse leptomeningeal seeding and local recurrence were found (vi, arrow heads). (F) Sequential expression of GFP fluorescence in NG‐27‐GCOA and NG‐65‐GCOA over 30 DAF indicating an area of a CO with dashed lines. *n* = 3 for each. Scale bars: 500 μm.

To investigate GBM invasion mechanisms, we established the GCOA model, achieved by bringing a GBM tumoroid into physical contact with a CO to form an assembloid. We adapted a previously published method for hiPSC‐derived COs [30] and patient‐derived GFP‐labeled GBM tumoroids [25, 29]. Two GBM samples were used (SNUH‐NG‐27 and SNUH‐NG‐65) and the concordance of the histological characteristics of the GBM tumoroids with their parental tumors was confirmed through H&E and IHC staining (Fig. S1a). The GFP labeling efficiency of the tumoroids was assessed by flow cytometry, which revealed efficiencies of approximately 83% for NG‐27 and 98% for NG‐65 (Fig. S1b). The COs at 40 DIV were characterized by their morphology, particularly the presence of cortical lobes and neural rosettes with markers of neuroepithelial cells and neurons (Fig. S1c,d). Subsequently, COs and GBM tumoroids were co‐cultured according to a previously described method for assembling assembloids (Fig. 1A) [31] [Correction added on 26 February 2025 after first online publication: The term “GBM tumors” has been replaced with “GBM tumoroids.”]. Briefly, one CO was placed together with a GBM tumoroid in a 1.5‐mL microcentrifuge tube, which fused within 3 days. The fusion area increased spontaneously at 7 DAF (Fig. 1B).

**Fig. 2:**
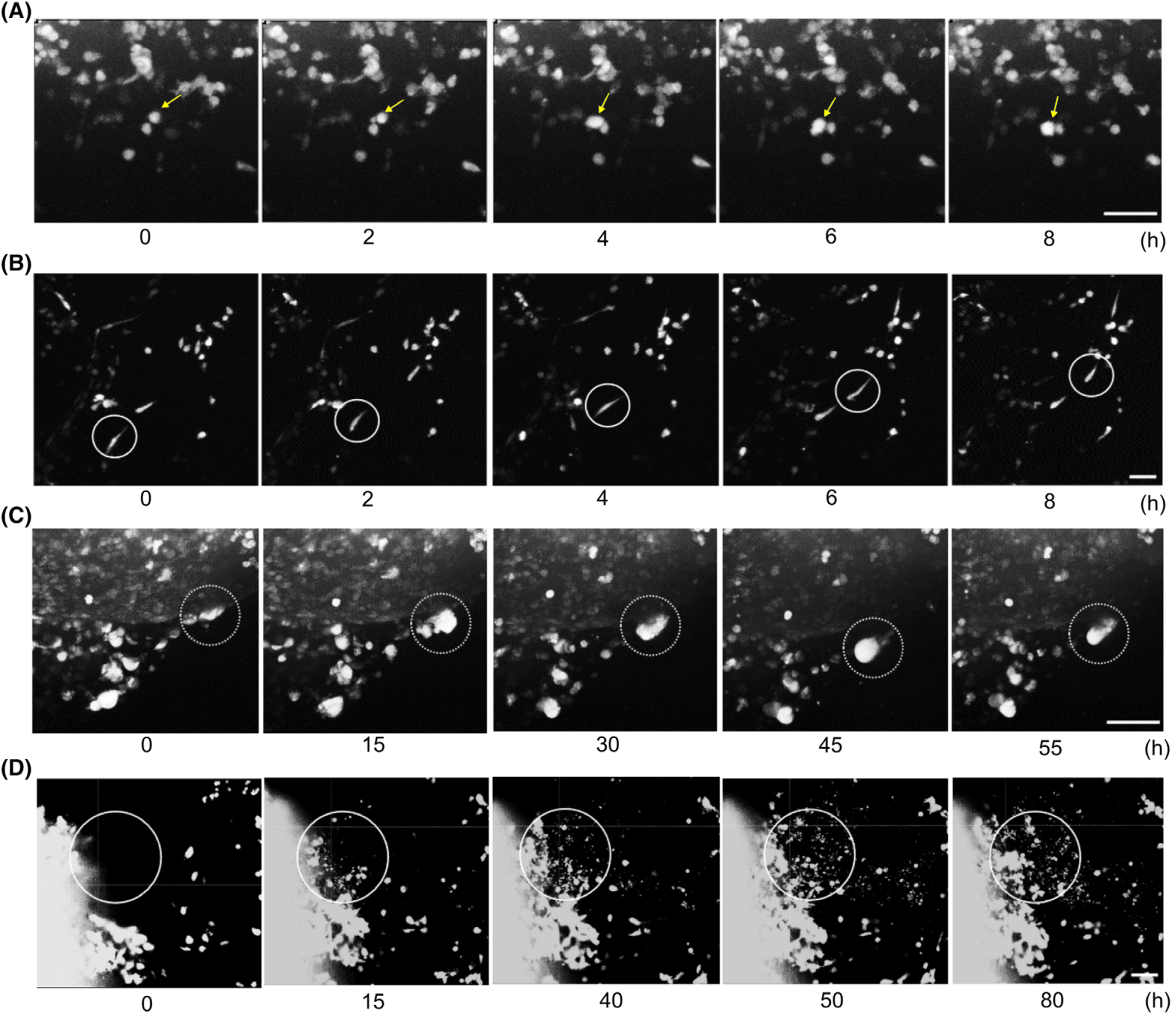
Different modes of invasion in GCOAs (Videos [Link], [Link]). Time‐lapse imaging of tumor cells in NG‐65‐GCOAs near the border between GBM tumoroids and cerebral organoids (COs). GBM, glioblastoma; GCOA, GBM‐cerebral organoid assembloid. Single‐cell invasion: amoeboid (A, yellow arrows) and mesenchymal (B, circles) for 8 h. Collective‐cell invasion: multicellular clusters (C, dotted circles) for 55 h and explosive invasion (D, circles) for 80 h. Scale bars: 100 μm. Videos are available as Videos [Link], [Link].

We monitored the movement and infiltration of fluorescently labeled tumor cells into the COs over 30 DAF using an ImageXpress Micro Confocal High‐Content Imaging System (HCS) (Fig. 1C‐1). Tumor cell invasion was quantified by comparing the relative area of normalized invaded GFP‐positive tumor cells in the CO to the total area of the CO (Fig. 1C‐2). The section with the widest infiltration area was selected and measured. Tumor cells began invading into the COs as early as 3 DAF. Scattered cells were observed at the leading edge of the invasion, whereas a denser aggregation of cells was observed closer to the tumoroid. By 10 DAF, tumor cells had invaded approximately 28% of the CO exhibiting extensive projections. This infiltration continued to progressively increase, reaching approximately 42% by 30 DAF (Fig. 1C). Owing to the limitations in visualizing the deep layers of GCOAs with HCS, whole‐mount staining and clearing of the GCOA was performed to visualize tumor cell invasion in depth. 3D topographical analyses and sections of GCOAs at different z‐stacks revealed the diffuse invasion of tumor cells into the CO at various depths. The tumor cells exhibited extensive infiltration, forming protrusions that were connected and invaded over long distances within the CO (Fig. 1D). IF staining was performed to assess the presence of immune cells in GCOAs. As anticipated for epithelial organoids, no immune cells were detected in the tumoroids or COs (Fig. S1e).

To assess whether the GCOA model reflects the invasion pattern of the parental tumors, we compared GCOAs from two patients (SNUH‐NG‐27 and SNUH‐NG‐65) with significantly different invasion potentials. A summary of the clinical and pathogenic data of the patients is presented in Table S1. Patient SNUH‐NG‐27 exhibited a clinical course typical of GBM. The patient had a solitary lesion with no apparent metastasis. After gross total resection (GTR) of the tumor and CCRT, the patient survived for 11 months. In contrast, patient SNUH‐NG‐65 exhibited an extremely aggressive clinical course. In addition to the main mass, there was a metastatic lesion in the suprasellar region. Despite undergoing GTR of the main mass, diffuse leptomeningeal seeding of the entire subarachnoid space of the brain and spine was observed 14 days postoperatively. The patient succumbed within 2 months of the initial diagnosis (Fig. 1E). Consistent with the behavior of the parental tumors, tumor cells in NG‐65‐GCOA demonstrated more aggressive and extensive invasion than those in NG‐27‐GCOA. In NG‐27‐GCOA, scattered tumor cells were observed at 10 DAF, while an invasion front region was observed within the CO at 20 DAF. Conversely, in NG‐65‐GCOA, the invasion front region was already apparent from at 3 DAF, and a significant number of tumor cells had infiltrated the far‐end region of the CO by 20 DAF (Fig. 1F). Given its highly invasive nature, NG‐65‐GCOA was selected for further *in vitro* experiments.

### 3.2 Revealing distinct invasion modes of tumor cells in GCOAs

As GCOAs enable intravital 3D‐imaging of tumor cells, we could monitor the dynamics of tumor cell invasion into the COs. The CO resembles the avascular region of the surrounding normal brain, the neuropil. Although perivascular invasion has been the primary focus when evaluating invasion in GBM, the neuropil is another key region of the brain in which tumor cells invade, thus emphasizing the relevance of the present model. The invasion of tumor cells into GCOAs was monitored by repetitive *in vitro* time‐lapse imaging for 90 h. This extensive observation facilitates the identification and characterization of various invasion patterns, including single‐ and collective‐cell invasion.

First, tumor cells within GCOAs exhibited individual movements resembling single‐cell invasion, with two distinct modes: amoeboid and mesenchymal. Single amoeboid cells displayed a morphologically uniform spherical shape and formed bleb‐like protrusions on the cell membrane, which assisted directional movement through contraction (Fig. 2A and Video S1). In contrast, mesenchymal single‐cell movement involved a continuous morphological transition from a round to an elongated spindle shape (Fig. 2B and Video S2).

Secondly, the tumor cells displayed collective‐cell invasion patterns as they invaded clusters. Multiple‐joined cells initiated invasion with a blunt bud‐like tip and several leader cells, while the rear cells adopted different morphologies during motion (Fig. 2C and Video S3). Within these multicellular clusters, the tumor cells exhibited morphological changes similar to those observed during cell division, indicating the possibility of simultaneous cell proliferation during invasion. Furthermore, we observed a unique phenomenon in which some cell clusters demonstrated an explosive collective movement, resulting in a group of cells breaking away from the main tumor mass (Fig. 2D and Video S4).

### 3.3 Formation of tumor microtube networks in GCOAs

**Fig. 3:**
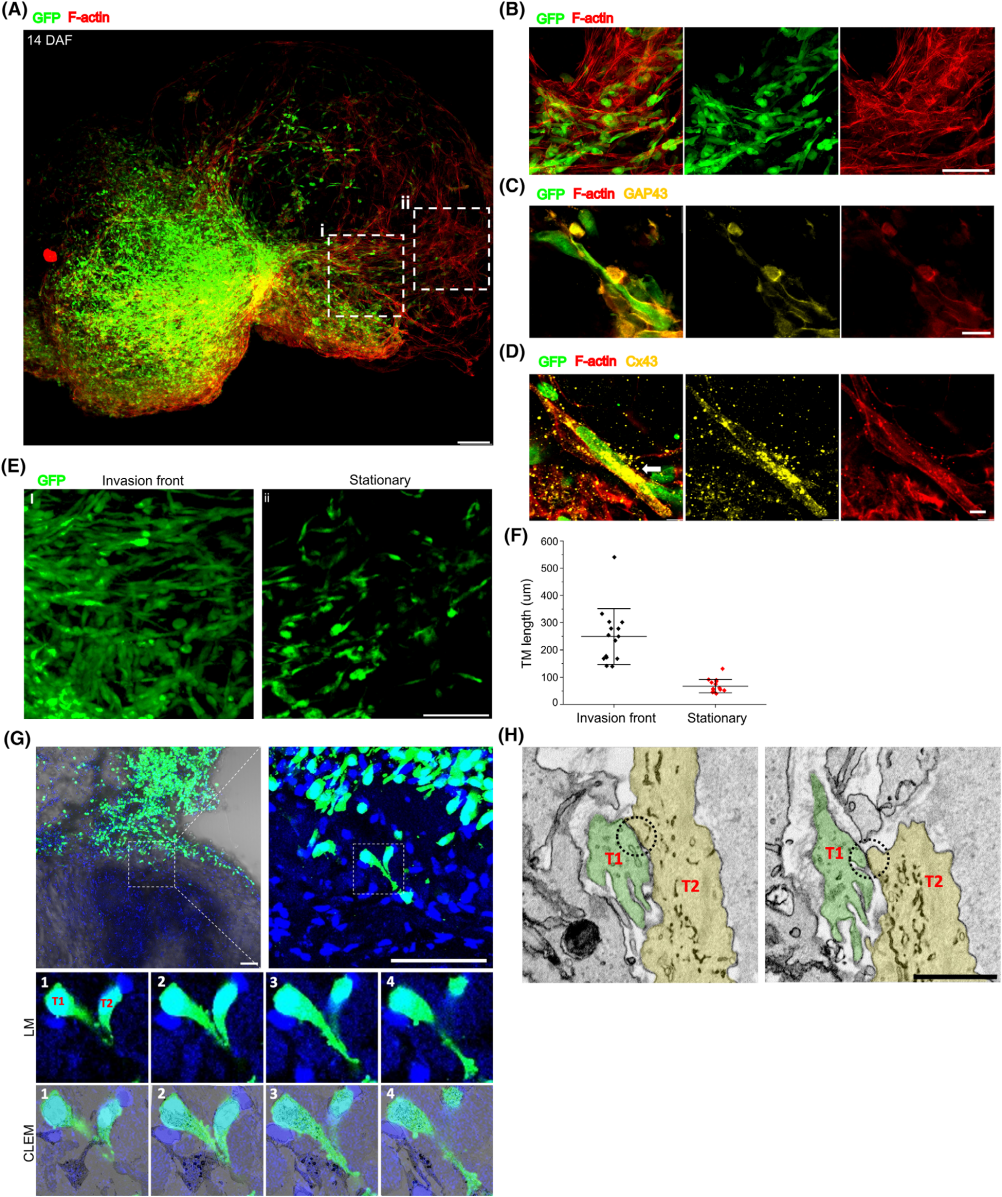
Characterization of tumor microtubes formed in GCOAs. (A) Whole‐mount staining image of the cleared NG‐65‐GCOA at 14 days after fusion (DAF), stained with F‐actin. *n* = 3. Scale bar: 100 μm. (B–D) Representative images of immunostaining for tumor microtube (TM) markers in NG‐65‐GCOA, F‐actin (B, whole‐mount staining), GAP43 (C, frozen‐sectioned staining), and Cx43 (D, whole‐mount staining) with an elevated expression where tumor cells are interacting (arrow). *n* = 20 (F‐actin) and 5 (GAP43 and Cx43). Scale bars: 100 μm (B) and 10 μm (C, D). (E) Representative images of GFP‐positive tumor cell infiltration with TMs at invasion front (i) and stationary (ii) tumor cell regions in A. *n* = 3. Scale bars: 50 μm. (F) Quantification of TM lengths in GFP‐positive tumor cells that formed in invasion front (i) and stationary (ii) tumor cell regions of the GCOA. *n* = 15 for each region; error bars are SD. (G) Representative correlative light and electron microscopy (CLEM) images of contacting GFP‐positive tumor cells (T1 and T2) with protrusions (dotted squares), using serial sectioning of NG‐65‐GCOA. The numbering of the serially sectioned images is indicated in the top left corners with a 2 μm distance between sections. *n* = 3. Scale bar: 100 μm. (H) Localization of tight junctions (dotted circles) between tumor cells T1 (green) and T2 (yellow) using serial sectioning of NG‐65‐GCOA with scanning electron microscopy (SEM). *n* = 3. Scale bar: 1 μm. GCOA, GBM‐cerebral organoid assembloid.

We then evaluated the multicellular tumor networks in GCOAs. Abundant long protruding membrane extensions were observed in the tumor cells inside the GBM tumoroid and CO (Fig. 3A). We examined whether these intercellular connections within the GCOA resembled TMs, which are recognized as direct intercellular communication routes in glioma networks [46]. First, we confirmed that the connections were rich in actin, a key feature of TMs (Fig. 3A,B). We also investigated the expression of growth‐associated protein 43 (GAP43) and connexin43 (Cx43), which have been reported as TM markers playing crucial roles in TM formation and interconnections, respectively [46]. Confocal microscopy revealed that GAP43‐positive tumor cells displayed long and continuous membranes with protrusion tips, similar to neural growth cones (Fig. 3C). Punctate labeling of Cx43 was also observed, particularly at sites where the tumor cells were in contact (Fig. 3D). Hence, the intercellular connections observed in GCOAs exhibit the characteristics of TMs.

We determined the length and number of TMs in GFP‐positive tumor cells at two distinct locations: near the border between the tumoroid and CO, representing the invasion front (Fig. 3A‐i), and in more distal regions of the CO, where the tumor cells may exhibit relatively stationary behavior (Fig. 3A‐ii). Measurements were averaged from at least three TMs from four randomly selected areas within each region. Cells at the invasion front exhibited longer TMs (average: 249 μm) than those in the stationary regions (average: 67 μm) (Fig. 3E,F).

A detailed ultrastructural analysis of the tube structures was performed using electron microscopy. CLEM involving serial sectioning of GCOAs combined with SEM confirmed that GFP‐positive tumor cells with TM‐like extended protrusions were not simply located adjacent to each other, but established actual cell‐to‐cell contacts (Fig. 3G). Notably, the contacts between tumor cells exhibited features consistent with tight junctions, implying clustered communication between neighboring cells (Fig. 3H).

### 3.4 Interaction between tumor and normal cells in GCOAs

**Fig. 4:**
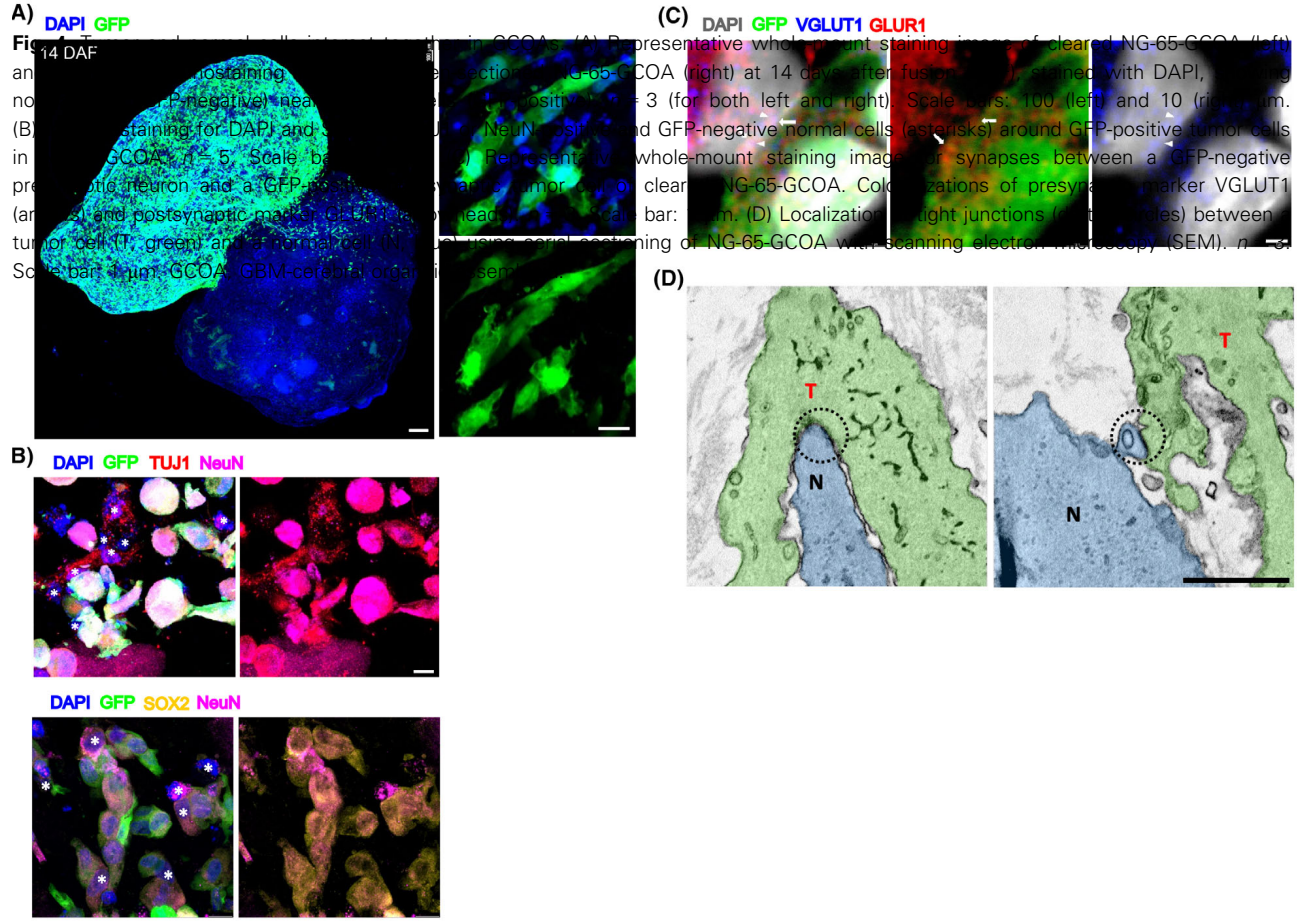
Tumor and normal cells interact together in GCOAs. (A) Representative whole‐mount staining image of cleared NG‐65‐GCOA (left) and enlarged immunostaining images of frozen‐sectioned NG‐65‐GCOA (right) at 14 days after fusion (DAF), stained with DAPI, showing normal cells (GFP‐negative) nearby tumor cells (GFP‐positive). *n* = 3 (for both left and right). Scale bars: 100 (left) and 10 (right) μm. (B) Immunostaining for DAPI and SOX2 or TUJ1 or NeuN‐positive and GFP‐negative normal cells (asterisks) around GFP‐positive tumor cells in NG‐65‐GCOA. *n* = 5. Scale bars: 10 μm. (C) Representative whole‐mount staining image for synapses between a GFP‐negative presynaptic neuron and a GFP‐positive postsynaptic tumor cell of cleared NG‐65‐GCOA. Colocalizations of presynaptic marker VGLUT1 (arrows) and postsynaptic marker GLUR1 (arrowheads). *n* = 3. Scale bar: 1 μm. (D) Localization of tight junctions (dotted circles) between a tumor cell (T, green) and a normal cell (N, blue) using serial sectioning of NG‐65‐GCOA with scanning electron microscopy (SEM). *n* = 3. Scale bar: 1 μm. GCOA, GBM‐cerebral organoid assembloid.

We conducted further investigations to explore the interactions between invading tumor cells and normal cells in GCOAs. Invading tumor cells were in close proximity to, and in direct contact with, resident normal cells within the COs (Fig. 4A). The CO portion of the GCOAs was rich in both NeuN‐ and TUJ1‐positive neurons, as well as SOX2‐positive neural progenitor cells, which were in contact with the invading tumor cells (Fig. 4B). Previous studies have demonstrated the formation of neurogliomal synapses (NGS) and their critical role in contributing to the malignant features of GBM [47, 48]. Because NGS typically form between glutamatergic presynaptic neurons and postsynaptic tumor cells, immunostaining was performed. Using super‐resolution confocal microscopy, we determined the postsynaptic density within a TM labeled with a glutamatergic AMPA receptor marker, glutamate receptor 1 (GLUR1). This postsynaptic marker corresponds to the excitatory presynaptic vesicle cluster labeled by vesicular glutamate transporter 1 (VGLUT1) with punctate patterns (Fig. 4C). Furthermore, we employed an image stack from serial section‐SEM to examine the contacts between tumor cells and neurons. We observed direct contact between the tumor and normal cells, suggesting synapse‐like junctions (Fig. 4D).

### 3.5 Differential gene expression and cellular states in invasive tumor cells

**Fig. 5:**
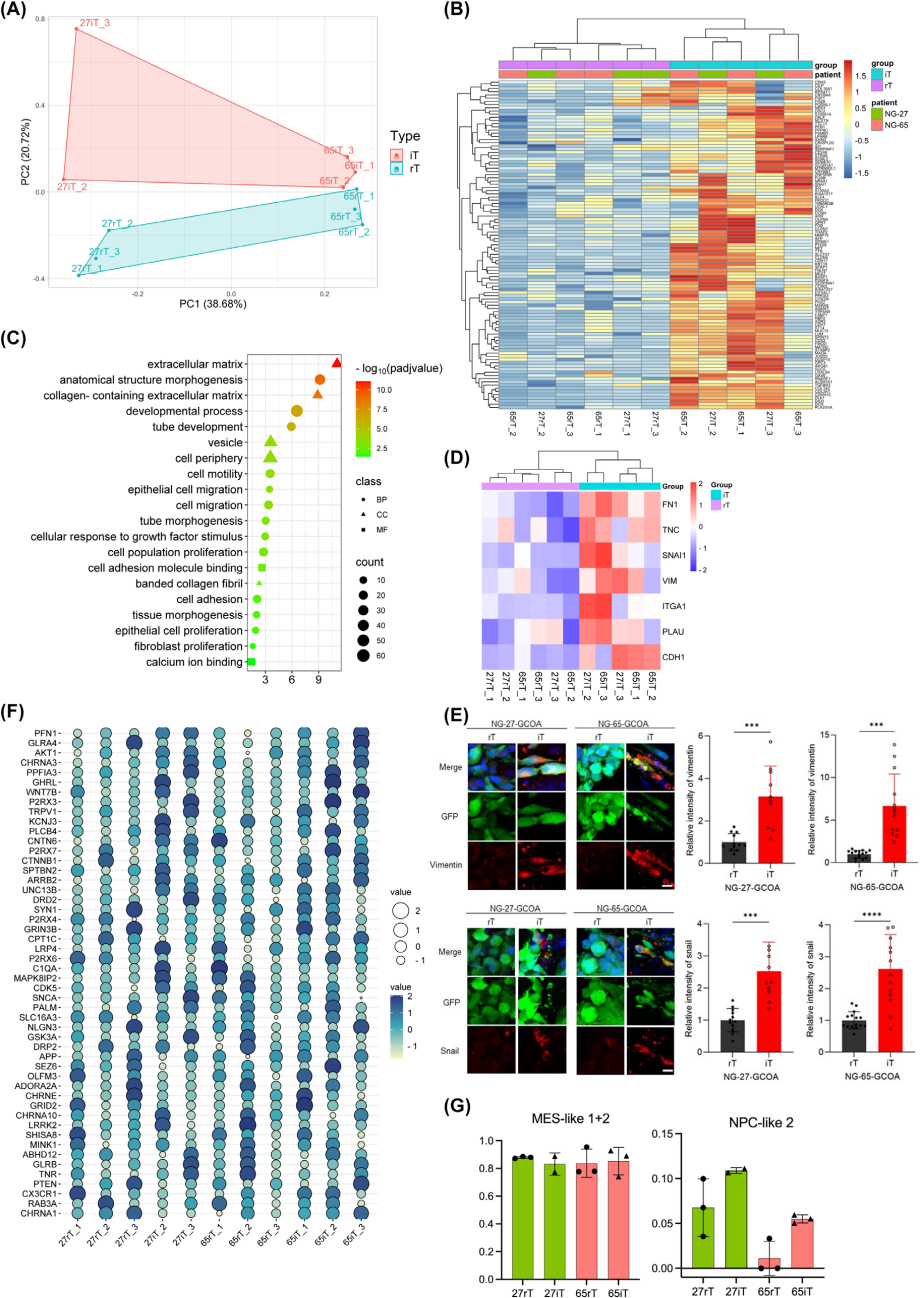
RNA sequencing analysis of invading tumor cells in GCOAs. (A) Unsupervised PCA clustering of rT and iT from two patient‐derived GBM‐cerebral organoid assembloid (GCOAs) (NG‐27‐GCOA and NG‐65‐GCOA) based on RNA quant‐seq data, with PC1 on the x‐axis and PC2 on the y‐axis. rT: tumor cells residing in the tumoroids of GCOAs (*n* = 3 for each), iT: tumor cells invading the cerebral organoid of GCOAs (*n* = 2 for NG‐27‐GCOA and *n* = 3 for NG‐65‐GCOA). PCA, principal component analysis. (B) Hierarchical clustering heatmap of 103 differentially expressed genes (DEGs) identified in iT (*n* = 2 for NG‐27‐GCOA and *n* = 3 for NG‐65‐GCOA) compared to rT (*n* = 3 for each) using DESeq2 [Correction added on 26 February 2025 after first online publication: DEG value has been replaced in this version.]. The selection criteria for DEGs include statistical significance of *P*‐value <0.05 and log_2_(fold change) >1. (C) GO analysis reveals statistically significant pathways of DEGs upregulated in iT (*n* = 2 for NG‐27‐GCOA and *n* = 3 for NG‐65‐GCOA) compared to rT (*n* = 3 for each) (Fig. S2b), with dots colored based on adjusted *P*‐values. BP, biological pathway; CC, cellular component; GO, gene ontology; MF, molecular function. (D) Heatmap depicting invasion marker genes expression in rT (*n* = 6) and iT (*n* = 5). The values are normalized to a z‐score, and hierarchical clustering is performed on columns. Red colors represent higher expression levels. Represented genes are *VIM*, *PLAU*, *SNAI1*, *TNC*, *FN1*, *ITGA1*, and *CDH1*. (E) Immunostaining for vimentin and snail, with quantification of the relative intensity of vimentin and snail in GFP‐positive tumor cells in iT and rT in NG‐27‐GCOA and NG‐65‐GCOA. Scale bars: 10 μm. *n* = 4 (vimentin) and *n* = 3 (snail); error bars represent SD. Significance values were calculated using Student's t‐test. ***P < 0.001 and ****P < 0.0001. (F) Dot plot depicting the expressions of synapse‐associated genes in rT (*n* = 6) and iT (*n* = 5) with values normalized to z‐score. Larger circles and deeper colors represent higher expression levels. (G) Bar plot generated from CIBERSORTx estimation, showing the combined fractions of MES‐like 1 and MES‐like 2, along with the NPC‐like 2 cellular states, within rT (*n* = 3) and iT (*n* = 2 for NG‐27‐GCOA and *n* = 3 for NG‐65‐GCOA) in NG‐27‐GCOA and NG‐65‐GCOA. Error bars represent SD. The *P*‐values for the estimation of the NPC‐like 2 state are 0.2 for NG‐27‐GCOA and 0.077 for NG‐65‐GCOA. MES, mesenchymal; NPC, neural progenitor cell. [Correction added on 26 February 2025 after first online publication: Figure 5 has been replaced in this version.]

We conducted RNA sequencing on sorted GFP‐positive tumor cells derived from GBM tumoroids (NG‐27 and NG‐65) and COs from GCOAs. The tumor cells within the GBM tumoroids of GCOAs were termed “rT” (resident tumor cells), while tumor cells in the COs were denoted as “iT” (invading tumor cells) (Fig. 1A). PCA results confirmed that samples from the same patient clustered together (PC1), whereas PC2 distinguished the gene expression patterns between the rT and iT of GCOAs (Fig. 5A and Fig. S2a).

DEG analysis was performed between iT and rT to determine transcriptomic differences between invasive and noninvasive tumor cells (Fig. S2b). We identified 103 DEGs that were significantly upregulated in iT (*P* < 0.05, log_2_(fold change) > 1, Table S4) [Correction added on 26 February 2025 after first online publication: The DEG value “106” has been corrected to “103.”]. The top‐scoring genes included collagen type 3 alpha 1 chain (*COL3A1*), neuropeptide Y (*NPY*), cyclin‐dependent kinase inhibitor 1C (*CDKN1C*), and decorin (*DCN*). *COL3A1* contributes to tumor infiltration and poor prognosis [49], whereas *NPY* plays a key role in tumor growth and progression [50]. The expression patterns of the DEGs were clustered based on iT and rT regardless of the tumor sample, suggesting that these genes reflect differences between invading and non‐invading cells (Fig. 5B). We investigated expression changes using qPCR and confirmed the RNA‐seq findings, which showed increased mRNA levels for the top‐scoring genes, including *DCN*, *CDKN1C*, *DIO3*, *NPY*, and *DLK1* in iT compared to those in rT (Fig. S2c). GO analysis of the 103 DEGs revealed significant enrichment in GO sets associated with anatomical structure development, cell adhesion, tube development, cell migration, cell motility, and extracellular matrix organization (Fig. 5C) [Correction added on 26 February 2025 after first online publication: The DEG value “106” has been corrected to “103.”]. Our results also revealed the upregulation of several key invasion‐related markers in the iT of both patient‐derived GCOAs, namely fibronectin 1 (*FN1*), tenascin C (*TNC*), snail family transcriptional repressor 1 (*SNAI1*), vimentin (*VIM*), integrin subunit alpha 1 (*ITGA1*), plasminogen activator, urokinase (*PLAU*), and cadherin_1 (*CDH1*) (Fig. 5D) [51, 52]. Additionally, the expression levels of vimentin and snail were higher in the iT than in the rT in both NG‐27‐GCOA and NG‐65‐GCOA, highlighting the invasive characteristics of iT in our model (Fig. 5E). Furthermore, the expression of synapse‐related genes was elevated in both rT and iT, further emphasizing the established roles of synaptic signals in tumor progression (Fig. 5F) [47, 48]. Consistent with previous reports underscoring the crucial role of glutamate receptors and cation channels, such as AMPAR, in GBM proliferation and invasion [47, 48], their expression was increased in rT and iT (Fig. S2d).

To further elucidate the clinical relevance of DEGs in iT compared with rT, we compared our results with the human GBM tissues RNA‐seq database established by the IVY‐Glioblastoma Atlas Project [44]. Specifically, we assessed the overlap between the upregulated gene expression patterns in iT and rT and those of the Leading Edge (LE) and Microvascular Proliferation (MVP) regions. The genes that overlapped with the LE DEGs included NPY, SPINT2, and ALDH1A1, which had log_2_(fold change) values of 6.64, 2.78 and 1.98, respectively (*P* < 0.05) [Correction added on 26 February 2025 after first online publication: The *P* values 6.72, 2.8, and 2.32 have been corrected to 6.64, 2.78 and 1.98.]. Similarly, genes overlapping with MVP DEGs included COL3A1, ID3, and DCN, which had log_2_(fold change) values of 2.38, 1.58, and 2.43, respectively (*P* < 0.05) [Correction added on 26 February 2025 after first online publication: The gene name “PPP1R14A” has been corrected to “ID3” and the *P* values 2.36, 2.54, and 3.72 have been corrected to 2.38, 1.58, and 2.43.]. These results indicated that tumor cells that acquire invasive behavior in GCOAs could be associated with clinically identified regions characterized by invasiveness or migration, even in the absence of vascular structures in GCOAs.

Multiple tumor cell states in tumor cells have been identified, and a recent landmark study proposed a four‐state model (neural‐progenitor (NPC)‐like, oligodendrocyte‐progenitor‐like, astrocyte‐like, and mesenchymal (MES)‐like) [53]. Given that various cell types undergo dynamic changes during disease progression and in response to treatment, we speculated on possible cell state changes as rT converts into iT. To estimate the cellular fractions of rT and iT, we used CIBERSORTx, an analytical web tool for imputing cell fractions within mixed cell populations [45] using scRNA‐seq data from the abovementioned study as the input reference file. In both patients, rT exhibited only small proportions of NPC‐like 2 states, while larger portions were observed in iT (10–11% in NG‐27 and 5% in NG‐65) (Fig. 5G and Table S5). [Correction added on 26 February 2025 after first online publication: This sentence has been rephrased in this version.]

## 4 Discussion

Cancer cell invasion is a hallmark of glioma progression. Cell–cell interactions and the TME have been identified as critical factors in studying the mechanism of glioma invasion. In this study, we established an assembloid model of patient‐derived GBM tumoroids and hiPSC‐derived COs that mimicked the invasion of tumor cells into the normal brain in a 3D architecture. GCOAs facilitated the real‐time visualization of tumor cell invasion, enabling the observation of various cell invasion patterns at a single‐cell resolution. Additionally, the structure of TM networks and interactions between tumor and normal cells were depicted. Comparing the RNA expression between rT and iT revealed the transcriptomic cues that contribute to phenotype alteration.

The highlight of our model is its ability to trace the real‐time movement of individual tumor cells within the normal brain milieu. Previous studies evaluating cell invasion dynamics were based on intravital imaging of tumor cells transplanted into rodent brains [54, 55]. However, these studies were limited by inadequate resolution, limited tissue penetration, and restricted imaging time windows [54, 56, 57]. Fortunately, our model captured the two primary categories of invasion: single‐cell and collective‐cell invasion utilizing time‐lapse imaging [58]. The visualization of collective‐cell invasion is particularly noteworthy because the diffuse infiltration of tumor cells has typically been investigated only in the context of single‐cell movement [3]. Collective‐cell invasion is characterized by a compact multicellular group of two or more neighboring cells. These groups have a defined actin‐rich leading edge with multiple mesenchymal‐like characteristics and sometimes exhibit blunt multicellular tips. This invasive strategy ultimately enables tumor cells to collectively remodel the extracellular matrix, thereby enhancing their invasive potential and promoting metastatic spread [59].

Furthermore, in contrast to previous studies that primarily focused on the perivascular migration of tumor cells [54, 60, 61], our model is one of the first 3D models to enable the evaluation of cell invasion in the avascular region. Diffuse infiltrative growth of tumor cells in the neuropil, an area with a minimal vascular structure, is a distinct feature of gliomas [3]. Since COs lack blood vessels, the GCOA model partially resembles the invasion of tumor cells into the avascular regions of the surrounding brain.

Recent studies have revealed a novel feature of GBM: TMs, which are elongated protrusions of tumor cells resembling dynamic axonal growth cones. These structures support cell infiltration, proliferation, and long‐distance communication via extensive intercellular networks [46, 62, 63]. Our GCOA model successfully established the formation of TM networks during tumor cell infiltration. We found that tumor cells at the invasion front, located at the border between the tumoroid and the CO, formed longer TMs than those farther away, resembling a less invasive state. This is contrary to a recent study that suggested that unconnected GBM cells are the primary drivers of invasion [64]. Differences in the experimental model or a lack of vascular structures may explain this discrepancy; however, further studies are required.

The formation of glutamatergic NGS between postsynaptic glioma cells and presynaptic neurons is a recent discovery, with its functional activity promoting the malignant behavior of GBM [47, 64]. We investigated the contact between tumor and normal cells in COs resembling surrounding brain tissue in our model. The colocalization of presynaptic markers in neurons and postsynaptic markers in TMs was detected by immunostaining. However, SEM exploration of neurons and tumor cells revealed only the presence of tight junctions and no signatures of chemical synaptic ultrastructure. Nevertheless, studies have shown the presence of perisynaptic contacts between neurons and tumor cells, suggesting that the structures observed in our study may be similar to these contacts [65, 66].

By sorting GFP‐positive tumor cells from the GBM tumoroids and COs of GCOAs, we were able to compare the transcriptomes of iT and rT. iT successfully demonstrated a transcriptome state favoring tumor invasion in our model, exhibiting an increase in well‐known invasion markers. The elevated expression of synapse‐related genes aligns with previous studies that highlighted the significance of the contact between presynaptic neurons and postsynaptic glioma cells in tumor progression [47, 48]. Moreover, a comparison of the proportions of cell states between rT and iT revealed an increase in the NPC‐like 2 state in iT. As the NPC‐like 2 state has been reported to be enriched in invasive tumor cells [64], this observation may reflect a cellular transition from rT to iT. Nonetheless, a single‐cell analysis with a significant number of cells is required to confirm this speculation.

In modeling tumor invasion, the importance of creating a TME accurately resembling that of a patient at cellular and structural heterogeneity levels cannot be overemphasized. In GBM, the constituents of the non‐neoplastic cell population are glial and immune cells. GBM is uniquely classified as an immunologically “cold” tumor as lymphocyte infiltration is limited and T lymphocytes are exhausted. The TME of GBM includes brain resident and infiltrating myeloid cells, dendritic cells, natural killer cells, and regulatory T‐cells [67]. The precise roles of resident microglia and tumor‐associated macrophages (TAMs) are under active research, however, it appears that TAMs present a tumor‐suppressive phenotype [68]. The unique GBM TME requires a preclinical model that recapitulates the immunocompetent environment, allowing for the successful development of immunotherapies. However, the GCOA model had the drawback of utilizing epithelial organoids that had the fundamental limitation of lacking immune cells. No traces of immune cells were found in either the tumoroids or COs using IF staining with antibodies for CD45 (leukocytes), CD3 (T lymphocytes), and CD68 (microglia) (Fig. S1d) as well as with gene signature analysis of immune cells in the RNA‐seq data (data not shown). As establishing a TME that includes the immune cell population is an active and urgent field of research, we plan on improving the current model by incorporating co‐cultures with various peripheral and/or tumor‐derived immune cell populations.

Our study has several limitations. First, the introduction of perfused vasculature and an immunologic niche into GCOAs is necessary to accurately recapitulate the human *in vivo* immune microenvironment. In addition, the investigation of NGS did not reveal chemical synapses such as synaptic vesicle clusters or synaptic clefts. Further electrophysiological studies would be able to provide evidence that supports the presence of NGS. Employing single‐cell sequencing or combining it with spatial context analysis may provide a more comprehensive understanding of GBM invasion. Finally, owing to the small number of patients, there were limitations to generalizing our results.

## 5 Conclusions

In summary, our methodology is the first 3D *in vitro* assembloid model that provides valuable insights into the features of human GBM invasion. Advancements achieved through the GCOA model may facilitate the exploration of invasion mechanisms and interactions between tumor cells and their microenvironment.

## Conflict of interest

The authors declare no competing interests.

## Author contributions

JK, RK, CKP, JIK, and JYL conceptualized the study, designed the experiments, and wrote the manuscript. JK performed *in vitro* experiments and analyzed the data. RK carried out bioinformatic experiments and analysis. WCL, KP, and SWI helped analyze bioinformatic data. WL, JSL, KHK, and JKW interpreted clinical MRI and cell invasion data. GHK and KJL performed the EM experiments. WL, SJ, YJL, GHK, KHK, WCL, KP, HJK, SWI, and KJL completed the data analysis. CKP, JIK, and JYL supervised the project. All authors reviewed and approved the manuscript, and contributed to the preparation of the manuscript.

## Supporting information


**Fig. S1.** Characterization of GBM tumoroids and cerebral organoids.
**Fig. S2.** Comparison of RNA sequencing data of invading and resident tumor cells in GCOAs.

**Table S1.** Summary of pathogenetic data and next‐generation sequencing genomic profiling of patients.
**Table S2.** Primers used for qPCR experiments.
**Table S3.** Quality control of sequencing data.
**Table S4.** Upregulated genes in invading tumor cells versus resident tumor cells.
**Table S5.** CIBERSORTx Results: Composition of GBM cellular states.

**Video S1.** Amoeboid single‐cell invasion.

**Video S2.** Mesenchymal single‐cell invasion.

**Video S3.** Collective cell invasion.

**Video S4.** Explosive cell invasion.

## Data Availability

All data necessary to evaluate the conclusions presented in this paper are provided within the manuscript or available in the Supplementary Materials. Furthermore, the raw sequencing data supporting this study's findings are publicly accessible in the NCBI Sequence Read Archive (SRA) database under accession code PRJNA#1013702.

## References

[1] Thakkar JP , Dolecek TA , Horbinski C , Ostrom QT , Lightner DD , Barnholtz‐Sloan JS , et al. Epidemiologic and molecular prognostic review of glioblastoma. Cancer Epidemiol Biomarkers Prev. 2014;23(10):1985–1996. 25053711 10.1158/1055-9965.EPI-14-0275 PMC4185005

[2] Stupp R , Mason WP , van den Bent MJ , Weller M , Fisher B , Taphoorn MJ , et al. Radiotherapy plus concomitant and adjuvant temozolomide for glioblastoma. N Engl J Med. 2005;352(10):987–996. 15758009 10.1056/NEJMoa043330

[3] Claes A , Idema AJ , Wesseling P . Diffuse glioma growth: a guerilla war. Acta Neuropathol. 2007;114(5):443–458. 17805551 10.1007/s00401-007-0293-7 PMC2039798

[4] Hou LC , Veeravagu A , Hsu AR , Tse VC . Recurrent glioblastoma multiforme: a review of natural history and management options. Neurosurg Focus. 2006;20(4):E5. 10.3171/foc.2006.20.4.2 16709036

[5] Kim J , Lee IH , Cho HJ , Park CK , Jung YS , Kim Y , et al. Spatiotemporal evolution of the primary glioblastoma genome. Cancer Cell. 2015;28(3):318–328. 26373279 10.1016/j.ccell.2015.07.013

[6] Perrin SL , Samuel MS , Koszyca B , Brown MP , Ebert LM , Oksdath M , et al. Glioblastoma heterogeneity and the tumour microenvironment: implications for preclinical research and development of new treatments. Biochem Soc Trans. 2019;47(2):625–638. 30902924 10.1042/BST20180444

[7] Vitorino P , Meyer T . Modular control of endothelial sheet migration. Genes Dev. 2008;22(23):3268–3281. 19056882 10.1101/gad.1725808 PMC2600767

[8] Yi Y , Hsieh IY , Huang X , Li J , Zhao W . Glioblastoma stem‐like cells: characteristics, microenvironment, and therapy. Front Pharmacol. 2016;7:477. 28003805 10.3389/fphar.2016.00477 PMC5141588

[9] Noushmehr H , Weisenberger DJ , Diefes K , Phillips HS , Pujara K , Berman BP , et al. Identification of a CpG Island methylator phenotype that defines a distinct subgroup of glioma. Cancer Cell. 2010;17(5):510–522. 20399149 10.1016/j.ccr.2010.03.017 PMC2872684

[10] Verhaak RG , Hoadley KA , Purdom E , Wang V , Qi Y , Wilkerson MD , et al. Integrated genomic analysis identifies clinically relevant subtypes of glioblastoma characterized by abnormalities in PDGFRA, IDH1, EGFR, and NF1. Cancer Cell. 2010;17(1):98–110. 20129251 10.1016/j.ccr.2009.12.020 PMC2818769

[11] Boccellato C , Rehm M . Glioblastoma, from disease understanding towards optimal cell‐based in vitro models. Cell Oncol (Dordr). 2022;45(4):527–541. 35763242 10.1007/s13402-022-00684-7 PMC9424171

[12] Paolillo M , Comincini S , Schinelli S . In vitro glioblastoma models: a journey into the third dimension. Cancers (Basel). 2021;13(10):2449. 34070023 10.3390/cancers13102449 PMC8157833

[13] Bigner DD , Bigner SH , Ponten J , Westermark B , Mahaley MS , Ruoslahti E , et al. Heterogeneity of genotypic and phenotypic characteristics of fifteen permanent cell lines derived from human gliomas. J Neuropathol Exp Neurol. 1981;40(3):201–229. 6260907 10.1097/00005072-198105000-00001

[14] Galli R , Binda E , Orfanelli U , Cipelletti B , Gritti A , De Vitis S , et al. Isolation and characterization of tumorigenic, stem‐like neural precursors from human glioblastoma. Cancer Res. 2004;64(19):7011–7021. 15466194 10.1158/0008-5472.CAN-04-1364

[15] Ben‐David U , Ha G , Tseng YY , Greenwald NF , Oh C , Shih J , et al. Patient‐derived xenografts undergo mouse‐specific tumor evolution. Nat Genet. 2017;49(11):1567–1575. 28991255 10.1038/ng.3967 PMC5659952

[16] Pine AR , Cirigliano SM , Nicholson JG , Hu Y , Linkous A , Miyaguchi K , et al. Tumor microenvironment is critical for the maintenance of cellular states found in primary glioblastomas. Cancer Discov. 2020;10(7):964–979. 32253265 10.1158/2159-8290.CD-20-0057 PMC10256258

[17] Kim J , Koo B‐K , Knoblich JA . Human organoids: model systems for human biology and medicine. Nat Rev Mol Cell Biol. 2020;21(10):571–584. 32636524 10.1038/s41580-020-0259-3 PMC7339799

[18] Clevers H . Modeling development and disease with organoids. Cell. 2016;165(7):1586–1597. 27315476 10.1016/j.cell.2016.05.082

[19] Pasca SP . The rise of three‐dimensional human brain cultures. Nature. 2018;553(7689):437–445. 29364288 10.1038/nature25032

[20] Klein E , Hau A‐C , Oudin A , Golebiewska A , Niclou SP . Glioblastoma organoids: pre‐clinical applications and challenges in the context of immunotherapy. Frontiers in Oncology. 2020;10:10. 33364198 10.3389/fonc.2020.604121 PMC7753120

[21] Drost J , Clevers H . Organoids in cancer research. Nat Rev Cancer. 2018;18(7):407–418. 29692415 10.1038/s41568-018-0007-6

[22] Azzarelli R . Organoid models of glioblastoma to study brain tumor stem cells. Front Cell Dev Biol. 2020;8:220. 32373607 10.3389/fcell.2020.00220 PMC7176979

[23] Bian S , Repic M , Guo Z , Kavirayani A , Burkard T , Bagley JA , et al. Genetically engineered cerebral organoids model brain tumor formation. Nat Methods. 2018;15(8):631–639. 30038414 10.1038/s41592-018-0070-7 PMC6071863

[24] Ogawa J , Pao GM , Shokhirev MN , Verma IM . Glioblastoma model using human cerebral organoids. Cell Rep. 2018;23(4):1220–1229. 29694897 10.1016/j.celrep.2018.03.105 PMC6892608

[25] Hubert CG , Rivera M , Spangler LC , Wu Q , Mack SC , Prager BC , et al. A three‐dimensional organoid culture system derived from human glioblastomas recapitulates the hypoxic gradients and cancer stem cell heterogeneity of tumors found in vivo. Cancer Res. 2016;76(8):2465–2477. 26896279 10.1158/0008-5472.CAN-15-2402 PMC4873351

[26] Jacob F , Salinas RD , Zhang DY , Nguyen PTT , Schnoll JG , Wong SZH , et al. A patient‐derived glioblastoma organoid model and biobank recapitulates inter‐ and intra‐tumoral heterogeneity. Cell. 2020;180(1):188–204.e22. 31883794 10.1016/j.cell.2019.11.036 PMC7556703

[27] Linkous A , Balamatsias D , Snuderl M , Edwards L , Miyaguchi K , Milner T , et al. Modeling patient‐derived glioblastoma with cerebral organoids. Cell Rep. 2019;26(12):3203–3211.e5. 30893594 10.1016/j.celrep.2019.02.063 PMC6625753

[28] Krieger TG , Tirier SM , Park J , Jechow K , Eisemann T , Peterziel H , et al. Modeling glioblastoma invasion using human brain organoids and single‐cell transcriptomics. Neuro‐Oncology. 2020;22(8):1138–1149. 32297954 10.1093/neuonc/noaa091 PMC7594554

[29] Kang RH , Park J , Kim J , Chowdhury T , Oh JH , Kim J , et al. A deep dive: SIWV tetra‐peptide enhancing the penetration of nanotherapeutics into the glioblastoma. ACS Biomater Sci Eng. 2022;8(10):4163–4174. 34196517 10.1021/acsbiomaterials.1c00653

[30] Lancaster MA , Corsini NS , Wolfinger S , Gustafson EH , Phillips AW , Burkard TR , et al. Guided self‐organization and cortical plate formation in human brain organoids. Nat Biotechnol. 2017;35(7):659–666. 28562594 10.1038/nbt.3906 PMC5824977

[31] Sloan SA , Andersen J , Pasca AM , Birey F , Pasca SP . Generation and assembly of human brain region‐specific three‐dimensional cultures. Nat Protoc. 2018;13(9):2062–2085. 30202107 10.1038/s41596-018-0032-7 PMC6597009

[32] Masselink W , Reumann D , Murawala P , Pasierbek P , Taniguchi Y , Bonnay F , et al. Broad applicability of a streamlined ethyl Cinnamate‐based clearing procedure. Development. 2019;146(3):dev166884. 30665888 10.1242/dev.166884 PMC7115989

[33] Kim J , Lee S , Lee J , Park JC , Kim KH , Ko JM , et al. Neurotoxicity of phenylalanine on human iPSC‐derived cerebral organoids. Mol Genet Metab. 2022;136(2):132–144. 35562278 10.1016/j.ymgme.2022.04.005

[34] Xu ZX , Kim GH , Tan JW , Riso AE , Sun Y , Xu EY , et al. Elevated protein synthesis in microglia causes autism‐like synaptic and behavioral aberrations. Nat Commun. 2020;11(1):1797. 32286273 10.1038/s41467-020-15530-3 PMC7156673

[35] Andrews S . FastQC: A Quality Control Tool for High Throughput Sequence Data. 2010.

[36] Smith THA , Sudbery I . UMI‐tools: modeling sequencing errors in unique molecular identifiers to improve quantification accuracy. Genome Res. 2017;27(3):491–499. 28100584 10.1101/gr.209601.116 PMC5340976

[37] Bushnell B . BBMap: a fast, accurate, splice‐aware aligner. Lawrence Berkeley National Laboratory; 2014.

[38] Dobin ADC , Schlesinger F , Drenkow J , Zaleski C , Jha S , Batut P , et al. STAR: ultrafast universal RNA‐seq aligner. Bioinformatics. 2013;29(1):15–21. 23104886 10.1093/bioinformatics/bts635 PMC3530905

[39] Danecek P , Bonfield JK , Liddle J , Marshall J , Ohan V , Pollard MO , et al. Twelve years of SAMtools and BCFtools. Gigascience. 2021;10(2):giab008. 33590861 10.1093/gigascience/giab008 PMC7931819

[40] Anders SPP , Huber W . HTSeq – a python framework to work with high‐throughput sequencing data. Bioinformatics. 2015;31(2):166–169. 25260700 10.1093/bioinformatics/btu638 PMC4287950

[41] Love MI , Wolfgang H , Anders S . Moderated estimation of fold change and dispersion for RNA‐seq data with DESeq2. Genome Biol. 2014;15:550. 25516281 10.1186/s13059-014-0550-8 PMC4302049

[42] Raudvere U , Kolberg L , Kuzmin I , Arak T , Adler P , Peterson H , et al. G:profiler: a web server for functional enrichment analysis and conversions of gene lists (2019 update). Nucleic Acids Res. 2019;47(W1):W191–W198. 31066453 10.1093/nar/gkz369 PMC6602461

[43] Ji X , Zhang H , Cui Q . A panel of synapse‐related genes as a biomarker for gliomas. Front Neurosci. 2020;14:822. 32848578 10.3389/fnins.2020.00822 PMC7431624

[44] Puchalski RBSN , Miller J , Dalley R , Nomura SR , Yoon JG . An anatomic transcriptional atlas of human glioblastoma. Science. 2018;360(6389):660–663. 29748285 10.1126/science.aaf2666 PMC6414061

[45] Newman AM , Steen CB , Liu CL . Determining cell type abundance and expression from bulk tissues with digital cytometry. Nat Biotechnol. 2019;37:773–782. 31061481 10.1038/s41587-019-0114-2 PMC6610714

[46] Osswald M , Jung E , Sahm F , Solecki G , Venkataramani V , Blaes J , et al. Brain tumour cells interconnect to a functional and resistant network. Nature. 2015;528(7580):93–98. 26536111 10.1038/nature16071

[47] Venkataramani V , Tanev DI , Strahle C . Glutamatergic synaptic input to glioma cells drives brain tumour progression. Nature. 2019;573:532–538. 31534219 10.1038/s41586-019-1564-x

[48] Venkatesh HS , Morishita W , Geraghty AC . Electrical and synaptic integration of glioma into neural circuits. Nature. 2019;573:539–545. 31534222 10.1038/s41586-019-1563-y PMC7038898

[49] Yuan LSB , Chen L , Qian K , Wang Y , Qian G , Zhu Y , et al. Overexpression of COL3A1 confers a poor prognosis in human bladder cancer identified by co‐expression analysis. Oncotarget. 2017;28(41):70508–70520. 10.18632/oncotarget.19733 PMC5642573 29050298

[50] Tilan JKJ . Neuropeptide Y (NPY) in tumor growth and progression: lessons learned from pediatric oncology. Neuropeptides. 2016;55:55–66. 26549645 10.1016/j.npep.2015.10.005 PMC4755837

[51] Gerashchenko TS , Novikov NM , Krakhmal NV , Zolotaryova SY , Zavyalova MV , Cherdyntseva NV , et al. Markers of cancer cell invasion: are they good enough? J Clin Med. 2019;8(8):1092. 31344926 10.3390/jcm8081092 PMC6723901

[52] Leung DHL , Phon BWS , Sivalingam M , Radhakrishnan AK , Kamarudin MNA . Regulation of EMT markers, extracellular matrix, and associated signalling pathways by long non‐coding RNAs in glioblastoma mesenchymal transition: a scoping review. Biology. 2023;12(6):818. 37372103 10.3390/biology12060818 PMC10294841

[53] Neftel JL C , Filbin MG , Hara T , Shore ME , Rahme GJ , Richman AR , et al. An integrative model of cellular states, plasticity, and genetics for glioblastoma. Cell. 2019;178:835–849. 31327527 10.1016/j.cell.2019.06.024 PMC6703186

[54] Alieva M , Leidgens V , Riemenschneider MJ , Klein CA , Hau P , van Rheenen J . Intravital imaging of glioma border morphology reveals distinctive cellular dynamics and contribution to tumor cell invasion. Sci Rep. 2019;9(1):2054. 30765850 10.1038/s41598-019-38625-4 PMC6375955

[55] Kienast Y , von Baumgarten L , Fuhrmann M , Klinkert WE , Goldbrunner R , Herms J , et al. Real‐time imaging reveals the single steps of brain metastasis formation. Nat Med. 2010;16(1):116–122. 20023634 10.1038/nm.2072

[56] Entenberg D , Oktay MH , Condeelis JS . Intravital imaging to study cancer progression and metastasis. Nat Rev Cancer. 2023;23(1):25–42. 36385560 10.1038/s41568-022-00527-5 PMC9912378

[57] Friedl P , Locker J , Sahai E , Segall JE . Classifying collective cancer cell invasion. Nat Cell Biol. 2012;14(8):777–783. 22854810 10.1038/ncb2548

[58] Pandya P , Orgaz JL , Sanz‐Moreno V . Modes of invasion during tumour dissemination. Mol Oncol. 2017;11(1):5–27. 28085224 10.1002/1878-0261.12019 PMC5423224

[59] Haeger A , Wolf K , Zegers MM , Friedl P . Collective cell migration: guidance principles and hierarchies. Trends Cell Biol. 2015;25(9):556–566. 26137890 10.1016/j.tcb.2015.06.003

[60] Tamura R , Miyoshi H , Sampetrean O , Shinozaki M , Morimoto Y , Iwasawa C , et al. Visualization of spatiotemporal dynamics of human glioma stem cell invasion. Mol Brain. 2019;12(1):45. 31060588 10.1186/s13041-019-0462-3 PMC6503361

[61] Farin A , Suzuki SO , Weiker M , Goldman JE , Bruce JN , Canoll P . Transplanted glioma cells migrate and proliferate on host brain vasculature: a dynamic analysis. Glia. 2006;53(8):799–808. 16541395 10.1002/glia.20334

[62] Weil S , Osswald M , Solecki G , Grosch J , Jung E , Lemke D , et al. Tumor microtubes convey resistance to surgical lesions and chemotherapy in gliomas. Neuro‐Oncology. 2017;19(10):1316–1326. 28419303 10.1093/neuonc/nox070 PMC5596180

[63] Roehlecke C , Schmidt MHH . Tunneling nanotubes and tumor microtubes in cancer. Cancers (Basel). 2020;12(4):857. 32244839 10.3390/cancers12040857 PMC7226329

[64] Venkataramani YY V , Schubert MC , Reyhan E , Tetzlaff SK , Wißmann N , Botz M , et al. Glioblastoma hijacks neuronal mechanisms for brain invasion. Cell. 2022;185:2899–2917. 35914528 10.1016/j.cell.2022.06.054

[65] Bernardinelli Y , Randall J , Janett E , Nikonenko I , Konig S , Jones EV , et al. Activity‐dependent structural plasticity of perisynaptic astrocytic domains promotes excitatory synapse stability. Curr Biol. 2014;24(15):1679–1688. 25042585 10.1016/j.cub.2014.06.025

[66] Yang Y , Wang XB , Frerking M , Zhou Q . Delivery of AMPA receptors to perisynaptic sites precedes the full expression of long‐term potentiation. Proc Natl Acad Sci USA. 2008;105(32):11388–11393. 18682558 10.1073/pnas.0802978105 PMC2496888

[67] Thorsson V , Gibbs DL , Brown SD , Wolf D , Bortone DS , Ou Yang TH , et al. The immune landscape of cancer. Immunity. 2018;48(4):812–830.e14. 29628290 10.1016/j.immuni.2018.03.023 PMC5982584

[68] Pires‐Afonso Y , Niclou SP , Michelucci A . Revealing and harnessing tumour‐associated microglia/macrophage heterogeneity in glioblastoma. Int J Mol Sci. 2020;21(3):689. 31973030 10.3390/ijms21030689 PMC7037936

