# Supplementary material for: Assembly of glioblastoma tumoroids and cerebral organoids: a 3D in vitro model for tumor cell invasion
Source: Mol Oncol. 2024 Oct 30;19(3):698–715. doi: 10.1002/1878-0261.13740 (PMC11887666; doi:10.1002/1878-0261.13740)
Supplement: Supplementary file 1 — Fig. S1. Characterization of GBM tumoroids and cerebral organoids. Fig. S2. Comparison of RNA sequencing data of invading and resident tumor cells in GCOAs. [file MOL2-19-698-s002.zip › mol213740-sup-0001-FiguresS1/Supplementary figure legends.docx]

**Figure S1. Characterization of GBM tumoroids and cerebral organoids.**

(a) Representative images of H&E staining and IHC staining for ATRX, c-MET, EGFR, GFAP and IDH1-R132H (IDH1mt) of primary tumor tissues and their tumoroids at the initiation of co-culturing. Scale bars: 20 μm on 400 magnification.

(b) Quantification of percentage of cells labeled with GFP in single-cell dissociated NG-27 and NG-65 (3 tumoroids pooled per line) measured by flow cytometry.

(c) Representative bright-field images of an CO at 40 days in vitro (DIV) covered by well-formed translucent cortical lobes (dotted square) (left), showing a cortical plate-like region (right). Scale bars: 1 mm (left) and 200 μm (right)

(d) Representative images of H&E staining and IF staining for the neural rosette of CO at 40 DIV (Nestin for neuroepithelial cells and TUJ1 for neurons). Scale bars: 20 μm on 400 magnification.

(e) Representative IF staining for immune cells (CD3, CD45 and CD68) of tumoroid, cerebral organoid (CO) at 40 DIV, GCOAs (NG-27-GCOA and NG-65-GCOA) and positive control (tonsil tissue). Scale bars: 10 μm (tumoroid, CO, positive control) and 20 μm (NG-27-GCOA and NG-65-GCOA) on 630 magnification.

**Figure S2. Comparison of RNA sequencing data of invading and resident tumor cells in GCOAs.**

(a) Unsupervised PCA clustering of of rT and iT from two patient-derived GCOAs (NG-27-GCOA and NG-65-GCOA), with PC2 (x-axis) and PC3 (y-axis).

(b) Volcano plot of DEG analysis of iT compared to rT, based on log_2_(fold change) (x-axis) (Relatively high expression of iT on the right and rT on the left) and log_2_(p-values) (y-axis). DEGs with statistical significance are annotated on top with a cut off of log_2_(fold change) of 1 and p-value of 0.05.

(c) Summary of qPCR validation of RNA-seq results showing increased mRNA levels of *DCN*, *CDKN1C*, *DIO3*, *NPY*, and *DLK1* in iT compared to rT. n = 3, except for iT in NG-27-GCOA (n= 2); error bars represent SEM.

(d) Balloon plot of the expression of *GRIA* genes - *GRIA1*, *GRIA2*, *GRIA3*, and *GRIA4 -* in rT and iT. The values indicate CPM count, represented by color and the size of the circles.
