# Supplementary figures and images for: Assembly of glioblastoma tumoroids and cerebral organoids: a 3D in vitro model for tumor cell invasion
Source: Mol Oncol. 2024 Oct 30;19(3):698–715. doi: 10.1002/1878-0261.13740 (PMC11887666; doi:10.1002/1878-0261.13740)

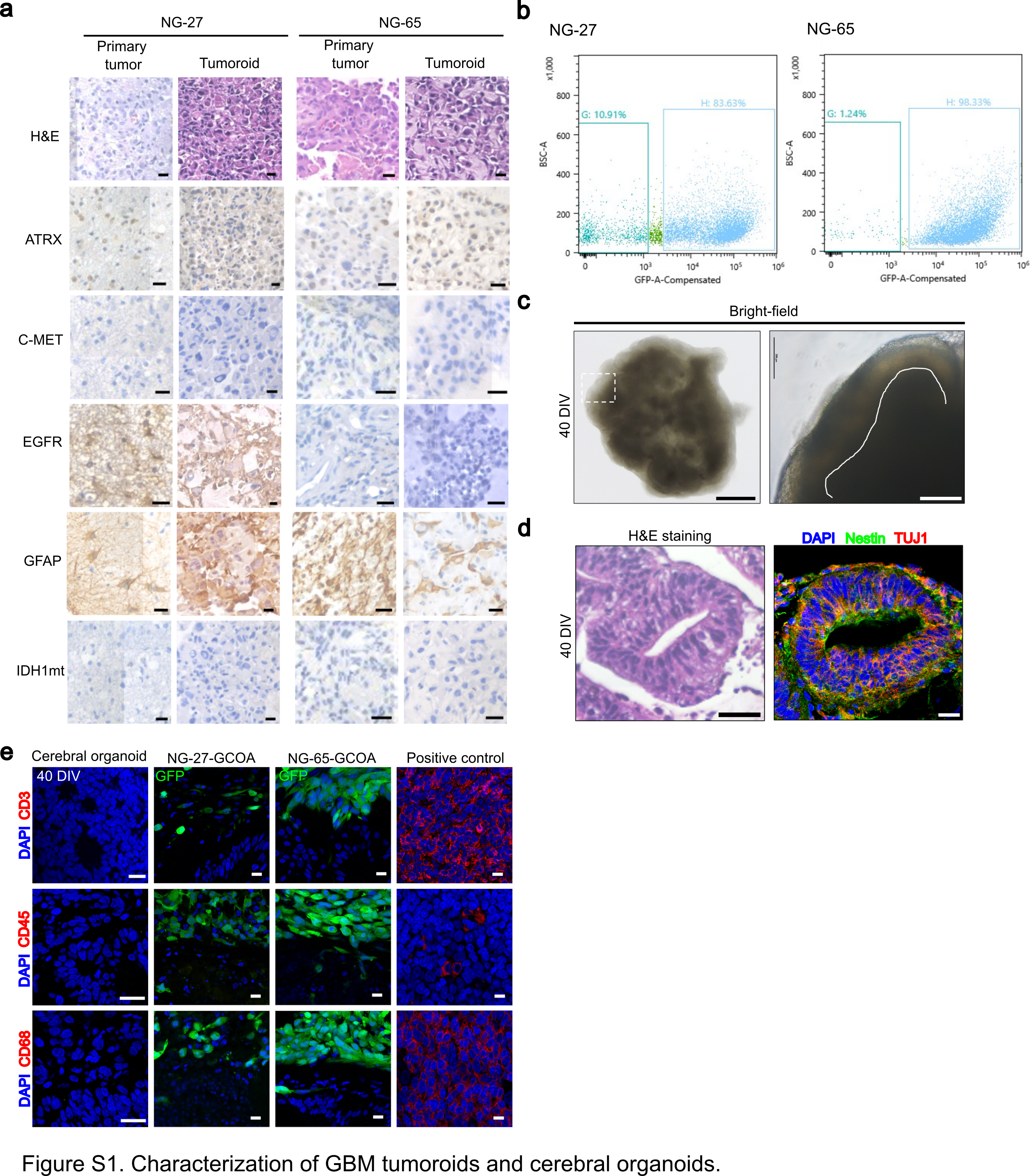

Supplement: Supplementary file 1 — Fig. S1. Characterization of GBM tumoroids and cerebral organoids. Fig. S2. Comparison of RNA sequencing data of invading and resident tumor cells in GCOAs. [file MOL2-19-698-s002.zip › mol213740-sup-0001-FiguresS1/240827_GCOA_figure_s1.tiff]

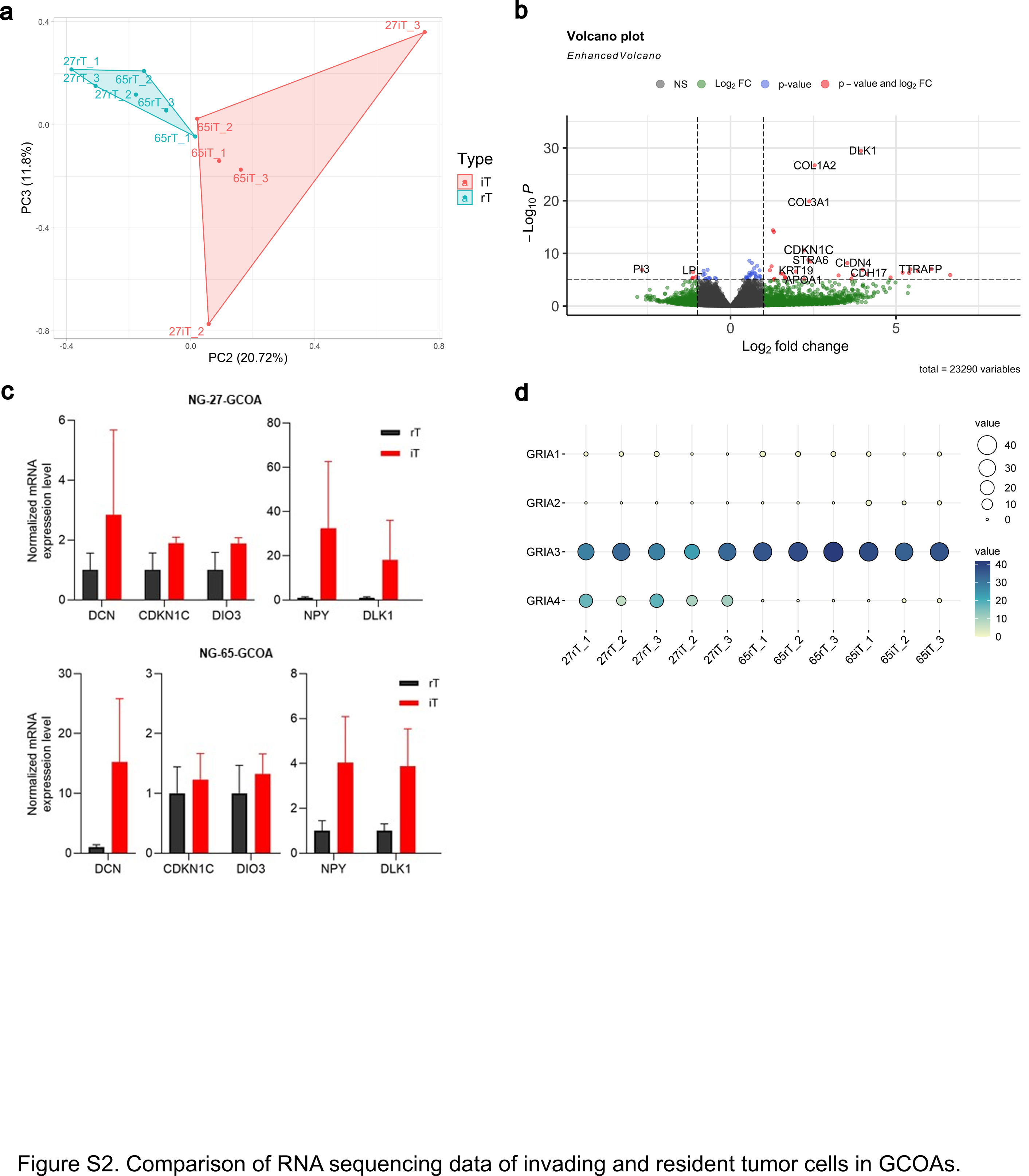

Supplement: Supplementary file 1 — Fig. S1. Characterization of GBM tumoroids and cerebral organoids. Fig. S2. Comparison of RNA sequencing data of invading and resident tumor cells in GCOAs. [file MOL2-19-698-s002.zip › mol213740-sup-0001-FiguresS1/240827_GCOA_figure_s2.tiff]
