# Supplementary material for: Assembly of glioblastoma tumoroids and cerebral organoids: a 3D in vitro model for tumor cell invasion
Source: Mol Oncol. 2024 Oct 30;19(3):698–715. doi: 10.1002/1878-0261.13740 (PMC11887666; doi:10.1002/1878-0261.13740)
Supplement: Supplementary file 2 — Table S1. Summary of pathogenetic data and next‐generation sequencing genomic profiling of patients. Table S2. Primers used for qPCR experiments. Table S3. Quality control of sequencing data. Table S4. Upregulated genes in invading tumor cells versus resident tumor cells. Table S5. CIBERSORTx Results: Composition of GBM cellular states. [file MOL2-19-698-s005.docx]

**Supplementary Table 1**

Summary of pathogenetic data and next-generation sequencing genomic profiling of patients

| **Category** | **SNUH-NG-27** | **SNUH-NG-65** |
| --- | --- | --- |
| Age | 49 years | 30 years |
| Gender | Male | Female |
| Initial Performance status (KPS) | 100 | 80 |
| Surgery | Yes (GTR^*^) | Yes (GTR) |
| Radiation Therapy | Yes | Yes* |
| Chemotherapy | Yes | Yes |
| Time to Recurrence | 7 months | 14 days |
| Survival duration | 11 months | 2 months |

KPS: Karnofsky Performance Scale

GTR: gross total resection

* The patient received palliative craniospinal irradiation

| **Patient** | **SNV/INDEL** | | | | | | | |
| --- | --- | --- | --- | --- | --- | --- | --- | --- |
|  | **Gene** | **Chr** | **Pos** | **Ref** | **Alt** | **AA** | **CDS** | **%Vaf** |
| SNUH-NG-27 | PIK3CA | chr3 | 178952085 | A | G | p.His1047Arg | c.3140A>G | 19.24 |
|  | TERT | chr5 | 1295228 | G | A | C228T | c.-124C>T | 22.83 |
|  | ERBB2 | chr17 | 37883207 | C | T | p.Pro1037Leu | c.3110C>T | 43.91 |
| SNUH-NG-65 | CDKN2C | chr1 | 51436168 | A | T | p.Gln43Leu | c.128A>T | 37.82 |
|  | TERT | chr5 | 1295228 | G | A | C228T | c.-124C>T | 48.48 |
|  | BRAF | chr7 | 140453136 | A | T | p.Val600Glu | c.1799T>A | 76.59 |
| **Patient** | **Translocation** | **Copy Number Alteration** | | | | | | |
|  |  | **Gene** | **Chr** | **Start** | **End** | **AvgL2R** | **CN** | **CNV** |
| SNUH-NG-27 | NA | CDKN2A | chr9 | 21967616 | 21995415 | -1.127 | 1 | Loss |
|  |  | CDKN2B | chr9 | 22002642 | 22009477 | -1.031 | 1 | Loss |
| SNUH-NG-65 | NA | CDKN2A | chr9 | 21967606 | 21995418 | -2.557 | 1 | Loss |
|  |  | CDKN2B | chr9 | 22002582 | 22009480 | -2.419 | 0 | Deletion |

**Supplementary Table 2**

Primers used for qPCR experiments.

| **Gene** | **Forward Primer** | **Reverse Primer** |
| --- | --- | --- |
| *DCN* | GCTCTCCTACATCCGCATTGCT | GTCCTTTCAGGCTAGCTGCATC |
| *CDKN1C* | AGATCAGCGCCTGAGAAGTCGT | TCGGGGCTCTTTGGGCTCTAAA |
| *DIO3* | GCCTACTTCGAGCGTCTCTATG | CATAGCGTTCCAACCAAGTGCG |
| *NPY* | AGGACGCACCAGCGGAGGAC | TGCAGGGTCTTCAAGCCGAGTTC |
| *DLK1* | CCCCAAAATGGATTCTGCGAGG | GGTTCTCCACAGAGTCCGTGAA |
| *GAPDH* | CATGAGAAGTATGACAACAGCCT | AGTCCTTCCACGATACCAAAGT |

**Supplementary Table 3**

Quality control of sequencing data

| **Tumor samples** | **FACS sorted cell count** | **RIN** | **Read count** |
| --- | --- | --- | --- |
| 27rT_1 | 197,718 | 8.5 | 2,207,557 |
| 27rT_2 | 145,709 | NA | 1,554,222 |
| 27rT_3 | 239,350 | 7 | 3,898,190 |
| 27iT_1 | 10,495 | NA | 67,000 |
| 27iT_2 | 63,191 | 7.3 | 613,410 |
| 27iT_3 | 29,301 | 5 | 368,907 |
| 65rT_1 | 308,769 | 8 | 4,149,001 |
| 65rT_2 | 703,653 | NA | 4,242,621 |
| 65rT_3 | 399,638 | 7.5 | 4,597,167 |
| 65iT_1 | 189,632 | 7 | 3,081,801 |
| 65iT_2 | 121,579 | 7.8 | 2,925,398 |
| 65iT_3 | 39,571 | 6.5 | 2,129,203 |

**Supplementary Table 4**

Upregulated genes in invading tumor cells versus resident tumor cells

| **Ensembl** | **Gene** | **Log2FoldChange** | **Negative logP** | **Score** |
| --- | --- | --- | --- | --- |
| ENSG00000185559 | *DLK1* | 3.941 | 29.46 | 116.114 |
| ENSG00000164692 | *COL1A2* | 2.541 | 26.708 | 67.861 |
| ENSG00000168542 | *COL3A1* | 2.38 | 19.876 | 47.3 |
| ENSG00000081051 | *AFP* | 6.07 | 7.023 | 42.632 |
| ENSG00000122585 | *NPY* | 6.637 | 5.901 | 39.166 |
| ENSG00000118271 | *TTR* | 5.434 | 6.964 | 37.846 |
| ENSG00000197406 | *DIO3* | 5.397 | 6.3 | 34 |
| ENSG00000171564 | *FGB* | 5.199 | 6.338 | 32.954 |
| ENSG00000189143 | *CLDN4* | 3.528 | 8.176 | 28.843 |
| ENSG00000104332 | *SFRP1* | 3.994 | 6.911 | 27.605 |
| ENSG00000079112 | *CDH17* | 4.119 | 6.093 | 25.095 |
| ENSG00000129757 | *CDKN1C* | 2.212 | 10.566 | 23.374 |
| ENSG00000165556 | *CDX2* | 3.697 | 5.906 | 21.838 |
| ENSG00000137868 | *STRA6* | 2.353 | 8.769 | 20.633 |
| ENSG00000011465 | *DCN* | 2.427 | 8.463 | 20.543 |
| ENSG00000122176 | *FMOD* | 3.262 | 5.817 | 18.977 |
| ENSG00000147257 | *GPC3* | 3.757 | 4.65 | 17.471 |
| ENSG00000134755 | *DSC2* | 3.671 | 4.639 | 17.031 |
| ENSG00000164266 | *SPINK1* | 3.702 | 4.095 | 15.159 |
| ENSG00000163586 | *FABP1* | 5.355 | 2.457 | 13.158 |
| ENSG00000171345 | *KRT19* | 1.971 | 6.556 | 12.925 |
| ENSG00000167642 | *SPINT2* | 2.775 | 4.342 | 12.051 |
| ENSG00000181885 | *CLDN7* | 3.059 | 3.822 | 11.693 |
| ENSG00000106541 | *AGR2* | 3.452 | 3.375 | 11.65 |
| ENSG00000127324 | *TSPAN8* | 3.832 | 3.031 | 11.614 |
| ENSG00000118137 | *APOA1* | 2.227 | 5.083 | 11.319 |
| ENSG00000124212 | *PTGIS* | 2.791 | 3.835 | 10.702 |
| ENSG00000155465 | *SLC7A7* | 2.322 | 4.443 | 10.317 |
| ENSG00000075223 | *SEMA3C* | 2.221 | 4.624 | 10.271 |
| ENSG00000174807 | *CD248* | 2.464 | 3.996 | 9.844 |
| ENSG00000117318 | *ID3* | 1.58 | 6.121 | 9.67 |
| ENSG00000077942 | *FBLN1* | 1.994 | 4.76 | 9.49 |
| ENSG00000179862 | *CITED4* | 1.65 | 5.387 | 8.888 |
| ENSG00000039068 | *CDH1* | 3.57 | 2.459 | 8.778 |
| ENSG00000149418 | *ST14* | 2.632 | 3.121 | 8.216 |
| ENSG00000123358 | *NR4A1* | 1.611 | 4.761 | 7.668 |
| ENSG00000114113 | *RBP2* | 3.858 | 1.978 | 7.63 |
| ENSG00000173702 | *MUC13* | 4.248 | 1.785 | 7.583 |
| ENSG00000165092 | *ALDH1A1* | 1.981 | 3.697 | 7.324 |
| ENSG00000176788 | *BASP1* | 2.081 | 3.377 | 7.025 |
| ENSG00000102837 | *OLFM4* | 4.169 | 1.67 | 6.961 |
| ENSG00000256618 | *MTRNR2L1* | 1.361 | 4.875 | 6.632 |
| ENSG00000125740 | *FOSB* | 1.301 | 5.009 | 6.518 |
| ENSG00000127990 | *SGCE* | 2.073 | 3.094 | 6.413 |
| ENSG00000132561 | *MATN2* | 1.786 | 3.416 | 6.101 |
| ENSG00000196754 | *S100A2* | 1.229 | 4.798 | 5.896 |
| ENSG00000136826 | *KLF4* | 2.083 | 2.585 | 5.385 |
| ENSG00000143643 | *TTC13* | 1.721 | 3.07 | 5.282 |
| ENSG00000197249 | *SERPINA1* | 1.438 | 3.593 | 5.166 |
| ENSG00000277791 | *PSMB3* | 1.365 | 3.56 | 4.857 |
| ENSG00000139329 | *LUM* | 1.387 | 3.395 | 4.709 |
| ENSG00000102098 | *SCML2* | 1.737 | 2.688 | 4.669 |
| ENSG00000253626 | *EIF5AL1* | 1.59 | 2.859 | 4.546 |
| ENSG00000189334 | *S100A14* | 1.891 | 2.355 | 4.455 |
| ENSG00000076944 | *STXBP2* | 1.429 | 3.072 | 4.39 |
| ENSG00000101331 | *CCM2L* | 1.199 | 3.61 | 4.329 |
| ENSG00000149923 | *PPP4C* | 1.554 | 2.757 | 4.284 |
| ENSG00000100122 | *CRYBB1* | 1.274 | 3.188 | 4.062 |
| ENSG00000007541 | *PIGQ* | 1.142 | 3.494 | 3.99 |
| ENSG00000120549 | *KIAA1217* | 1.718 | 2.301 | 3.954 |
| ENSG00000139679 | *LPAR6* | 1.184 | 3.331 | 3.945 |
| ENSG00000125968 | *ID1* | 1.05 | 3.706 | 3.893 |
| ENSG00000116711 | *PLA2G4A* | 1.52 | 2.531 | 3.848 |
| ENSG00000108518 | *PFN1* | 1.391 | 2.72 | 3.782 |
| ENSG00000171747 | *LGALS4* | 2.145 | 1.673 | 3.587 |
| ENSG00000196431 | *CRYBA4* | 1.129 | 3.132 | 3.535 |
| ENSG00000132386 | *SERPINF1* | 1.23 | 2.811 | 3.458 |
| ENSG00000217555 | *CKLF* | 1.045 | 2.988 | 3.122 |
| ENSG00000149021 | *SCGB1A1* | 1.882 | 1.65 | 3.106 |
| ENSG00000069702 | *TGFBR3* | 1.309 | 2.255 | 2.952 |
| ENSG00000084636 | *COL16A1* | 1.437 | 2.048 | 2.942 |
| ENSG00000189221 | *MAOA* | 1.448 | 2.015 | 2.918 |
| ENSG00000129824 | *RPS4Y1* | 1.363 | 2.091 | 2.849 |
| ENSG00000162576 | *MXRA8* | 1.228 | 2.307 | 2.832 |
| ENSG00000162998 | *FRZB* | 1.424 | 1.976 | 2.813 |
| ENSG00000162878 | *PKDCC* | 1.008 | 2.756 | 2.779 |
| ENSG00000062038 | *CDH3* | 1.573 | 1.76 | 2.769 |
| ENSG00000136098 | *NEK3* | 1.535 | 1.617 | 2.482 |
| ENSG00000106484 | *MEST* | 1.404 | 1.749 | 2.456 |
| ENSG00000114656 | *KIAA1257* | 1.06 | 2.194 | 2.327 |
| ENSG00000103485 | *QPRT* | 1.208 | 1.918 | 2.318 |
| ENSG00000137216 | *TMEM63B* | 1.322 | 1.751 | 2.315 |
| ENSG00000156136 | *DCK* | 1.027 | 2.25 | 2.311 |
| ENSG00000162736 | *NCSTN* | 1.135 | 2.014 | 2.286 |
| ENSG00000149599 | *DUSP15* | 1.115 | 2.003 | 2.233 |
| ENSG00000161677 | *JOSD2* | 1.12 | 1.873 | 2.097 |
| ENSG00000132000 | *PODNL1* | 1.257 | 1.656 | 2.082 |
| ENSG00000168646 | *AXIN2* | 1.323 | 1.567 | 2.073 |
| ENSG00000198276 | *UCKL1* | 1.006 | 2.055 | 2.066 |
| ENSG00000183087 | *GAS6* | 1.366 | 1.5 | 2.049 |
| ENSG00000113578 | *FGF1* | 1.137 | 1.696 | 1.928 |
| ENSG00000129538 | *RNASE1* | 1.304 | 1.469 | 1.916 |
| ENSG00000124216 | *SNAI1* | 1.145 | 1.663 | 1.904 |
| ENSG00000103196 | *CRISPLD2* | 1.141 | 1.661 | 1.894 |
| ENSG00000134480 | *CCNH* | 1.003 | 1.826 | 1.831 |
| ENSG00000245680 | *ZNF585B* | 1.175 | 1.553 | 1.825 |
| ENSG00000197461 | *PDGFA* | 1.223 | 1.461 | 1.787 |
| ENSG00000106546 | *AHR* | 1.065 | 1.664 | 1.772 |
| ENSG00000177380 | *PPFIA3* | 1.168 | 1.515 | 1.769 |
| ENSG00000102996 | *MMP15* | 1.031 | 1.678 | 1.73 |
| ENSG00000173451 | *THAP2* | 1.007 | 1.668 | 1.68 |
| ENSG00000116771 | *AGMAT* | 1.021 | 1.611 | 1.645 |
| ENSG00000138615 | *CILP* | 1.113 | 1.449 | 1.614 |

**Supplementary Table 5**

CIBERSORTx Results: Composition of GBM cellular states

| Mixture | AClike | MESlike1 | MESlike2 | NPClike1 | NPClike2 | OPClike | P-value | Correlation | RMSE |
| --- | --- | --- | --- | --- | --- | --- | --- | --- | --- |
| 27rT_1 | 0 | 0.609 | 0.278 | 0.046 | 0.068 | 0 | 0.02 | 0.552 | 0.95 |
| 27rT_2 | 0 | 0.601 | 0.271 | 0 | 0.1 | 0.028 | 0.012 | 0.581 | 0.918 |
| 27rT_3 | 0 | 0.602 | 0.278 | 0.084 | 0.036 | 0 | 0.008 | 0.596 | 0.904 |
| 27iT_2 | 0 | 0.688 | 0.201 | 0 | 0.111 | 0 | 0.016 | 0.56 | 0.988 |
| 27iT_3 | 0 | 0.716 | 0.058 | 0.119 | 0.107 | 0 | 0.012 | 0.582 | 0.958 |
| 65rT_1 | 0 | 0.326 | 0.454 | 0.22 | 0 | 0 | 0.04 | 0.524 | 0.865 |
| 65rT_2 | 0 | 0.306 | 0.471 | 0.223 | 0 | 0 | 0.008 | 0.599 | 0.808 |
| 65rT_3 | 0 | 0.532 | 0.423 | 0 | 0.033 | 0.012 | 0.02 | 0.553 | 0.933 |
| 65iT_1 | 0 | 0.409 | 0.482 | 0.054 | 0.055 | 0 | 0.03 | 0.538 | 0.892 |
| 65iT_2 | 0 | 0.383 | 0.357 | 0.209 | 0.051 | 0 | 0.046 | 0.508 | 0.881 |
| 65iT_3 | 0 | 0.539 | 0.39 | 0.012 | 0.06 | 0 | 0.048 | 0.505 | 0.97 |
